# Self-Reported Trait Mindfulness and Affective Reactivity: A Motivational Approach Using Multiple Psychophysiological Measures

**DOI:** 10.1371/journal.pone.0119466

**Published:** 2015-03-06

**Authors:** Danielle Cosme, Stefan Wiens

**Affiliations:** 1 Department of Psychology, Stockholm University, Stockholm, Sweden; 2 Department of Psychology, University of Oregon, Eugene, Oregon, United States of America; Vanderbilt University, UNITED STATES

## Abstract

As a form of attention, mindfulness is qualitatively receptive and non-reactive, and is thought to facilitate adaptive emotional responding. One suggested mechanism is that mindfulness facilitates disengagement from an affective stimulus and thereby decreases affective reactivity. However, mindfulness has been conceptualized as a state, intervention, and trait. Because evidence is mixed as to whether self-reported trait mindfulness decreases affective reactivity, we used a multi-method approach to study the relationship between individual differences in self-reported trait mindfulness and electrocortical, electrodermal, electromyographic, and self-reported responses to emotional pictures. Specifically, while participants (N = 51) passively viewed pleasant, neutral, and unpleasant IAPS pictures, we recorded high-density (128 channels) electrocortical, electrodermal, and electromyographic data to the pictures as well as to acoustic startle probes presented during the pictures. Afterwards, participants rated their subjective valence and arousal while viewing the pictures again. If trait mindfulness spontaneously reduces general emotional reactivity, then for individuals reporting high rather than low mindfulness, response differences between emotional and neutral pictures would show relatively decreased early posterior negativity (EPN) and late positive potential (LPP) amplitudes, decreased skin conductance responses, and decreased subjective ratings for valence and arousal. High mindfulness would also be associated with decreased emotional modulation of startle eyeblink and P3 amplitudes. Although results showed clear effects of emotion on the dependent measures, in general, mindfulness did not moderate these effects. For most measures, effect sizes were small with rather narrow confidence intervals. These data do not support the hypothesis that individual differences in self-reported trait mindfulness are related to spontaneous emotional responses during picture viewing.

## Introduction

Scientific interest in mindfulness and mindfulness-based therapies to alleviate psychological distress and promote well-being has increased exponentially over the past 15 years. Mindfulness is a form of attention that is rooted in the present moment and is qualitatively receptive and non-reactive, and as such it is thought to facilitate emotion regulation [1]. In line with this, randomized controlled trials using the mindfulness-based intervention Mindfulness-Based Stress Reduction (MBSR) [2] found that MBSR reduced symptoms of psychological distress, such as self-reported anxiety, depression, and stress, and improved self-reported positive affect, self-compassion, and satisfaction with life (for a review, see [3]). MBSR has also been associated with positive outcomes of emotion regulatory processes, such as reduced rumination [4] and decreased activation in brain regions implicated in self-referential processing and elaboration [5]. In addition, experienced meditators have reported lower levels of rumination and fewer difficulties in emotion regulation, compared to non-meditators [6]. Similarly, self-reported mindfulness has been positively correlated with self-reported use of adaptive emotion regulation strategies [7] and negatively correlated with self-reported difficulty in emotion regulation [8]. Together these findings support the notion that mindfulness is related to adaptive emotional responding, but the underlying mechanisms are not clearly understood.

One suggested mechanism is that mindfulness facilitates disengagement from the affect-relevant stimulus and thereby decreases affective reactivity (i.e., one’s reaction in response to affect-relevant events) [9]. An affective response is characterized by several physiological components, including: threshold for reactivity, peak amplitude of response, rise time to peak, and recovery time [10]. Paying attention mindfully is proposed to facilitate disengagement from an emotion-provoking stimulus by attenuating the peak amplitude and speeding up recovery, thereby decreasing the overall emotional response [9]. This is in contrast to continued engagement with the stimulus, which may promote elaborative self-referential processing and rumination, processes which are associated with psychological distress [11].

Despite the proposed link between mindfulness and affective reactivity, mindfulness is a rich concept originating in ancient Buddhist traditions that is difficult to succinctly and adequately operationalize [12]. Within the context of Western science, mindfulness is often defined as the awareness generated through “paying attention in a particular way: on purpose, in the present moment, and non-judgmentally” (p. 4) [13]. Within the psychological literature, it is described as consisting of two components: self-regulation of attention, and orientation to one’s current experience with an attitude of curiosity, acceptance, and openness [14]. These two components are thought to work synergistically to facilitate disengagement from affect-relevant stimuli, leading to decreased reactivity [14].

Self-regulation of attention helps practitioners to focus in the present moment and to observe their current experience, including thoughts, feelings, perceptions, and bodily sensations. As attentional resources are limited, focusing attention on the current experience inhibits elaboration, rumination, and other self-referential processes [14]. That is not to say that experiences (thoughts, feelings, perceptions) are suppressed, but rather they are merely noted as mental events and not elaborated on further, keeping attention on current experience. This focus of attention in the present moment might also facilitate disengagement by increasing awareness of one's initial reactivity to a stimulus, allowing one to let go and return to the present moment [15].

The second component of mindfulness describes a particular orientation of curiosity, openness, and non-judgmentalness to all experiences. This attitude of acceptance of current experience, regardless of valence or desirability, is thought to reduce avoidant behaviors and facilitate decoupling of stimulus-response reactivity through exposure, such as in exposure therapy [15]. In addition, open observation of one’s experience can create space between the observer and their thoughts, feelings, and perceptions. Rather than seeing them as accurate judgments or reflections of self, they may be observed as transitory mental events that do not have to be identified with [14]. This distance during exposure is likely to facilitate disengagement as it weakens the typical, spontaneous emotional response tendencies to affect-relevant stimuli.

In sum, being mindful may facilitate disengagement by maintaining attention in the present moment, thereby inhibiting elaborative processes, and by maintaining an attitude of acceptance, thereby creating distance between the stimulus and response that weakens habitual emotional response tendencies. Thus, mindfulness is thought to facilitate the development of dispositional nonreactivity [9]. Several studies have investigated effects of mindful states or mindfulness interventions on responses to emotional pictures. Results have supported the link between mindfulness and decreased reactivity to emotional pictures [16–18]. Arch and Craske [16] employed a 15-minute focused breathing induction and found that, compared to inductions of worry or unfocused attention, focused breathing was associated with decreased self-reported negative affect after passively viewing aversive pictures and greater willingness to engage with unpleasant pictures in a subsequent task. Ortner and colleagues [17] investigated effects of a 7-week mindfulness intervention on emotional responding. Participants were shown pleasant, unpleasant, and neutral pictures and performed two tasks. The first task was an emotional interference task (categorizing tones as high or low while viewing emotional and neutral pictures), and the second was a passive viewing task. Skin conductance responses and self-reported emotional intensity were collected as measures of affective reactivity. Mindfulness training was associated with decreased (post-intervention vs. pre-intervention) emotional interference while viewing unpleasant pictures, decreased self-reported emotional intensity while viewing pleasant and unpleasant pictures, and decreased skin conductance responses to pleasant and unpleasant pictures. Lastly, Taylor and colleagues [18] investigated mindful attention versus uninstructed attention to pleasant, unpleasant, and neutral pictures in novice and experienced meditators, and used self-reported ratings of emotional intensity after each picture as a measure of affective reactivity. For both novice and experienced meditators, mindful attention decreased self-reported emotional intensity to pleasant, neutral, and unpleasant pictures. Together, these findings support the hypothesis that mindfulness, when operationalized as a state or intervention, decreases affective reactivity while viewing emotional pictures.

However, in the field of psychology, the term mindfulness is currently used in three ways: to describe a *state*, as in a mode of awareness entered during mindfulness meditation, an *intervention*, in which participants learn mindfulness meditation, and a *trait*, implying a stable quality that differs between individuals. Effects for mindfulness may differ depending on how it is conceptualized. The studies reviewed above [16–18] provide evidence of the relationship between mindfulness treated as a state or intervention and decreased affective reactivity to emotional pictures. In contrast, there is mixed evidence from studies on affective reactivity during passive picture viewing when mindfulness is treated a self-reported trait [19–21].

Ostafin and colleagues [19] found evidence supporting the relationship between self-reported trait mindfulness and reduced self-reported and behavioral measures of affective reactivity. Self-reported trait mindfulness was measured with the Acting with awareness and Non-judging of inner experience facets of the Five Facet Mindfulness Questionnaire (FFMQ) [22]. High self-reported trait mindfulness was associated with lower self-reported negative affect and accessibility of aversive words after a negative emotion induction using unpleasant pictures. In contrast, Sauer and colleagues [20] used a large sample (*N* = 247) and found no relationship between self-reported trait mindfulness and ratings of unpleasant and neutral pictures, or word ratings after aversive evaluative conditioning.

Brown and colleagues [21] used evoked brain potentials (the late positive potential; LPP) as a measure of affective reactivity. They found that high self-reported trait mindfulness on the Mindful Attention Awareness Scale (MAAS) [23] was associated with low LPP amplitudes while viewing unpleasant pictures. Although this finding supports the idea that self-reported trait mindfulness reduces affective reactivity, other research employed similar designs but compared meditators and non-meditators directly rather than using self-reported trait mindfulness as an independent measure and did not replicate this finding [24, 25]. Because experienced meditators should ostensibly be more mindful than non-meditators, one would expect to find clear differences in LPP responses as a function of meditation experience. However, the available evidence is inconclusive. On the one hand, Sobolewski and colleagues [25] found experienced Buddhist meditators to have attenuated LPP amplitudes for unpleasant pictures, but the effect was found at left and right frontal electrodes rather than at centro-parietal sites where it is generally reported [26]. On the other hand, using a group of yogic meditators practicing in the Sudarshan Kriya tradition, Gootjes and colleagues [24] found no difference in LPP amplitudes to emotional pictures between meditators and non-meditators during a passive viewing task. While there are no obvious reasons for these inconclusive results for LPP in active meditators, it is nonetheless important to replicate findings by Brown et al. [21] and to even extend them, if possible. If it can be shown that trait mindfulness is associated with reduced LPP responses to emotional pictures versus neutral pictures (rather than just to unpleasant pictures per se), then these results would provide convincing evidence for a specific effect of trait mindfulness on affective reactivity. In sum, the current evidence that self-reported trait mindfulness is associated with decreased affective reactivity to emotional pictures is rather limited and mixed.

The aim of the present study was to further investigate the relationship between self-reported trait mindfulness and affective reactivity to emotional pictures during passive picture viewing using multiple measures that comprehensively characterize affective reactivity. Participants rated trait mindfulness using two commonly used self-report scales, the MAAS [23] and FFMQ [22]. We used both scales as they are thought to measure different aspects of mindfulness and thus, they allowed us to separate the components thought to be related to self-regulation of attention (MAAS and the Acting with awareness subscale on the FFMQ) and attitude of acceptance (the subscales of Non-judging of inner experience and of Nonreactivity to inner experience on the FFMQ). In the study, participants completed a passive viewing task, looking at pleasant, unpleasant, and neutral pictures while electroencephalography (EEG), electromyography (EMG), and electrodermal measures were recorded. Participants heard sporadic loud bursts of white noise to evoke a startle response. Self-reported ratings of valence and arousal were collected while participants viewed the pictures a second time.

In the present study, emotion was characterized in terms of valence and arousal. Emotion can be conceptualized in terms of discrete categories (e.g., fear, disgust, etc.) or in term of the motivational dimensions of valence and arousal [27]. According to the motivational model, unpleasant and pleasant pictures differ in their valence, and both unpleasant and pleasant pictures differ from neutral pictures in their arousal level. Because research has demonstrated clear effects of valence and arousal on different physiological and self-report measures [27], the present study adopted a motivational perspective on emotion. Further, because various dependent measures differ in their sensitivity to either valence or arousal [27], the predictions and data analyses were focused on either the valence effect (i.e., pleasant vs. unpleasant) or the arousal effect (i.e., pleasant + unpleasant combined vs. neutral) of emotion.

To index valence effects, we measured self-reported valence ratings to the pictures and startle eyeblink responses during the pictures. The startle response is characterized in humans by muscular contractions in the torso and face, and indexes the activation of the defensive system [28]. Startle response amplitude is modulated by one’s affective state and can be conveniently recorded by measuring the amplitude of the eyeblink response to the startle probe [29]. The eyeblink amplitude, which is potentiated during negative affective states and attenuated during positive affective states, has been shown to be modulated particularly while viewing highly arousing emotional pictures [27]. Therefore, an effect of picture valence was predicted such that pleasant pictures would elicit more pleasant self-reported valence ratings than unpleasant pictures, whereas unpleasant pictures would elicit greater startle responses than pleasant pictures. Self-reported trait mindfulness was expected to moderate these effects with higher mindfulness being associated with smaller differences in arousal effects on valence ratings and startle responses.

To index arousal effects (i.e., pleasant + unpleasant combined vs. neutral), we measured self-reported arousal to the pictures, skin conductance responses to picture onset, and three event-related potentials derived from the EEG: the early posterior negativity (EPN) and the late positive potential (LPP) to picture onset, and the P3 to startle onset. The skin conductance response (SCR) is regulated by the autonomic nervous system with sympathetic innervation of the sweat glands producing the skin conductance response [30]. As such, SCR can serve as a measure of sympathetic arousal [31]. The EPN is a relative negativity over occipito-temporal electrodes to arousing versus neutral pictures about 200 ms after picture onset. It appears to reflect a call for attentional resources [32]. The LPP is a positive deflection of the ERP waveform occurring at centro-parietal sites approximately 400 ms after picture onset. Although it may already be observed to neutral pictures per se, arousal effects on the LPP are apparent in the difference ERP waveforms to arousing versus neutral pictures [33]. This relative LPP is thought to index greater attention allocation to arousing pictures than to neutral pictures [34]. As such, EPN and LPP were used to measure the call, as well as the actual allocation, of attention to arousing versus neutral pictures. Lastly, the P3 component, which is a centro-parietal positivity approximately 300 ms after stimulus onset, indexes attention allocation and has been used in affect-modulated startle response paradigms to assess the relative allocation of attention to the startle probes versus pictures [35]. Specifically, the P3 to startle probes is reduced during viewing of arousing (unpleasant or pleasant) pictures versus neutral pictures [36, 37]. This is consistent with the idea that more attentional resources are drawn to arousing pictures than to neutral pictures and thus, that fewer attentional resources are available to process the additional startle probe during arousing than neutral pictures. Therefore, across participants, we expected to find a general effect of picture arousal on P3 amplitudes, with smaller P3 amplitudes to startle probes presented on trials in which arousing pictures were shown than when neutral pictures were shown. In sum, as a consequence of arousal effects, responses were expected to be greater for arousing than neutral pictures for self-reported arousal, skin conductance responses, and LPP. In contrast, responses were expected to be lower (i.e., less positive) for arousing than neutral pictures for the EPN and P3. Self-reported trait mindfulness was predicted to moderate these response differences with higher mindfulness being associated with relatively smaller response differences, indicating decreased affective reactivity.

## Methods

### Participants

A convenience sample of 56 students and professionals (30 women and 26 men) was recruited via the psychology department at Stockholm University and through an online recruitment website. Two participants were excluded because they reported moderate to severe levels of depression on the Beck Depression Inventory (BDI) [38]. Three additional participants were excluded due to technical errors (*n* = 1), excessively noisy EEG signal (*n* = 1), and missing survey data (*n* = 1), yielding a total of 51 participants in the study (25 women, 26 men). Participants were between the ages of 19 and 51 (*M* = 25, *SD* = 6.8), were fluent in both Swedish and English, were not currently being treated for any psychological disorders, and had normal or corrected to normal vision. Seven participants (14%) had some meditation experience, ranging from 1–8 years of regular practice (*M* = 3.1). Mean trait anxiety on the State-Trait Anxiety Inventory (STAI-T) [39] was 36.5 (*SD* = 7.5) and mean depression scores (BDI) were 4.6 (*SD* = 4.0). The study was approved by the Stockholm section of the Central Ethical Review Board in Sweden and was conducted in accordance with the guidelines in the Helsinki Declaration. All participants gave written informed consent, were debriefed after the experiment, and were compensated with course credit or 2 movie vouchers.

### Self-Reported Measures

**Mindfulness.** Self-reported trait mindfulness was assessed using two self-report scales: the Mindfulness Awareness Attention Scale (MAAS) [23] and the Five Facet Mindfulness Questionnaire (FFMQ) [22]. The MAAS is a 15-item scale that assesses present-moment awareness, a core facet of mindfulness, as a dispositional trait. An example item is “I could be experiencing some emotion and not be conscious of it until some time later” and answers are scored on a 6-point scale ranging from almost always to almost never. All items are reverse-scored and higher scores indicate higher mindfulness (possible range = 15–90; sample α = .85).

The FFMQ is a 39-item scale measuring five aspects of trait mindfulness [22]. These include: Observing (e.g., “I notice the smells and aromas of things.”), Describing (e.g., “I’m good at finding words to describe my feelings.”), Acting with awareness (FFMQ-AWA; e.g., “I am easily distracted.”—reversed item), Non-judging of inner experience (FFMQ-NJ; e.g., “I tell myself I shouldn’t be feeling the way I’m feeling.”—reversed item), and Nonreactivity to inner experience (FFMQ-NR; e.g., “I perceive my feelings and emotions without having to react to them.”). Answers are scored using a 5-point scale ranging from *never or very rarely true* to *very often or always true*. Higher scores indicate higher mindfulness (possible range = 39–195; sample psychometrics: entire scale α = .88, Observing α = .71, Describing α = .87, Acting α = .85, Non-judging α = .85, Nonreacting α = .77).

Notably, we administered the English versions of the MAAS and the FFMQ as they have been used extensively in research whereas the alternative Swedish versions have not. In addition, the Swedish version of the FFMQ has 10 less items and a different factor structure than the English version [40]. Although all participants reported fluency in English, they were given the option to answer each question with “I do not understand the question” to avoid language-related confounds. Consequently, these items were coded as missing data and the means for each participant were calculated excluding these items. While no items were missing on the FFMQ-AWA, 4 participants had no more than 2 items missing on the MAAS, 3 participants had no more than 2 items missing on the FFMQ-NJ, and 13 participants had no more than 4 items missing on the FFMQ-NR.

**Trait Anxiety.** Trait anxiety was measured using the State-Trait Anxiety Inventory (STAI-T) [39]. The STAI-T consists of 20 items measuring trait anxiety and answers are scored on a 4-point scale ranging from *not at all* (scored as 1) to *very much so* (scored as 4). An example item is “I worry too much over something that really doesn’t matter.” Higher scores indicate higher anxiety (possible range = 20–80; sample α = .85).

**Depression.** Depression was measured using the Beck Depression Inventory (BDI) [38]. The BDI is a 21-item questionnaire measuring attitudes and symptoms of depression. Each item is scored with increasing severity of the symptom on a scale from 0–3. An example item is: 0 = *I do not feel sad*, 1 = *I feel sad*, 2 = *I am sad all the time and can’t snap out of it*, 3 = *I am so sad and unhappy I can’t stand it*. Higher scores indicate higher depression, with scores greater than 21 indicating moderate to severe clinical depression (possible range = 0–63; sample α = .75).

### Materials and Presentation

Stimuli were 72 pictures (see S1 Table) from the International Affective Picture System (IAPS) [41]. The pictures varied in valence and arousal, and emotional pictures (pleasant and unpleasant) were selected to be highly arousing. The pictures included 24 pleasant pictures depicting highly arousing pleasant images (e.g., babies, erotica; valence *M* = 7.4, *SD* = 0.66, arousal *M* = 5.6, *SD* = 1.1), 24 unpleasant pictures depicting highly arousing unpleasant images (e.g., mutilation, threat; valence *M* = 2.1, *SD* = 0.57, arousal *M* = 6.3, *SD* = 0.63), and 24 neutral pictures (e.g., neutral faces, mushrooms; valence *M* = 5.2, *SD* = 0.38, arousal *M* = 3.4, *SD* = 0.31). For normative valence and arousal ratings separately, independent-samples *t* tests of the ratings (with pictures as subjects) showed that the three picture categories differed from each other (*p* < .001).

Subjective affective experience was measured using the Self-Assessment Manikin (SAM) [42]. This scale consists of two 9-point (from 1 to 9) scales assessing how pleasant/unpleasant and how aroused/calm participants felt while viewing each of the 72 pictures. Higher scores on the valence scale indicated greater positive affect, whereas higher scores on the arousal scale indicated greater arousal.

The pictures were presented on a 21″ View Sonic p227f CRT-screen with a refresh rate of 100 Hz and a resolution of 1024 x 768 pixels. The viewing distance was 70 cm and was maintained with a chin rest. The picture size was 19.0 x 14.2 cm and the visual angle was 15.4 x 11.6 degrees. The experiment was programmed in Presentation 14.8 (Neurobehavioral Systems, Albany, CA).

### Procedure

Before being accepted into the experiment, participants were screened for psychological disorders through an online survey. In this survey, participants provided information about background and demographic variables, and completed a battery of psychological scales (as described in ‘Self-reported measures’). After survey data were collected and screened, accepted participants came to the lab and participated in the 2-hour psychophysiological experiment. After written informed consent was given, EEG, EMG, and skin conductance electrodes were attached. Participants were then instructed to passively view the 72 pleasant, unpleasant, and neutral pictures, which were presented in a quasi-random order (no more than 2 pictures of the same valence category in a row) that was generated for each participant. As in a previous study [43], each picture was displayed at fixation for 6 seconds followed by a randomized interval of 4–12 seconds (i.e., inter-trial interval was 10–18 seconds, *M* = 14 s) during which a fixation cross remained on the screen. A burst of white noise (square wave with a sudden onset at 102 dB for 50 ms) was presented on each trial (1500 ms, 4500 ms, or 7500 ms after picture onset) through headphones to elicit the startle response. The task was divided into 2 blocks (36 trials each), and participants were allowed to take a break between blocks. After the passive viewing task was completed, participants completed another task (not reported here), and then viewed the pictures once again, rating their subjective feelings of pleasantness/unpleasantness and arousal for each picture. Finally participants were debriefed, thanked, and compensated.

### Physiological data collection

EEG data were collected from 128 channels using an Active Two BioSemi system (BioSemi, Amsterdam, Netherlands). A BioSemi electrode cap was used with electrodes placed according to the ABC layout—that is, electrodes were positioned in increasingly larger circles from the vertex (http://www.biosemi.com/download/Cap_coords_all.xls). The electrodes CMS and DRL (approximately at CP1 and CP2) were used as reference and ground electrodes, respectively. Data were sampled at 2048 Hz and filtered with a hardware low-pass filter at 400 Hz. Facial EMG was measured with a pair of 4 mm BioSemi Ag-AgCl electrodes placed on the left orbicularis oculi muscle, according to the guidelines from Blumenthal and colleagues [29]. Skin conductance was measured with a pair of 1 cm BioSemi Ag-AgCl electrodes placed on the palm of the left hand. All electrodes were filled with electrolyte conductance gel (SIGNAGEL, Parker Laboratories, Inc., Fairfield, NJ, USA). All physiological data was processed offline using MATLAB (version R2013b, The MathWorks Inc., Natick, Massachusetts, http://www.mathworks.com).

### Data reduction

**SCR**. The continuous raw data for skin conductance were high-pass filtered at 0.05 Hz with a 4th order Butterworth filter and low-pass filtered at 10 Hz with a 4th order Butterworth filter. After filtering, these data were downsampled to 100 Hz. Then, epochs were extracted between 0 and 7 seconds after picture onset. Trials in which the startle probe was presented 1500 ms after picture onset were excluded to avoid confounding of the SCR to the pictures with the SCR to the startle. Thus, the total number of trials was 48: for pleasant, neutral, and unpleasant pictures each, there were 8 trials with startles at 4500 ms and 8 trials at 7500 ms. Each epoch was baseline corrected with a baseline interval of 0–900 ms after picture onset. Scoring was conducted according to guidelines [30]. Individual trials were visually inspected using a response window of 1–4 seconds after picture onset, and the response onset and peak were selected manually within this window. This procedure was used to ensure that only true SCR responses were selected, and to avoid incorrect automatic selection due to, for example, noise that exceeded a specified threshold (e.g., 0.05 μS) or incorrect selection on trials with two peaks. To avoid any scoring biases, the scorers were blind to both the trait mindfulness scores of each subject and also to the picture condition of individual trials. Trials with no perceptible response were scored as non-responses (zero) but were included when calculating SCR magnitude. Trials with excessive noise were rejected and not included in the magnitude calculation. Of the 49 participants with SCR recordings, 9 participants had no more than 9 (19% of 48) missing trials. SCR values were log transformed (log[SCR+1]) to normalize the data and then magnitude scores (the mean across all included trials) were calculated for each condition.

**EMG.** Startle eyeblink EMG was processed in accordance with guidelines [29]. First, the continuous raw EMG data were high-pass filtered at 28 Hz using a 4th order Butterworth filter and rectified. Next, trial epochs were extracted (between 200 ms before and 200 ms after startle onset) and low-pass filtered at 40 Hz with a 4th order Butterworth filter. The data were then baseline corrected, with a baseline window between 50 and 0 ms before startle onset. The maximum amplitude during the response window (20–150 ms after stimulus onset) was automatically extracted. Trials with a response amplitude that exceeded mean baseline plus 3 times the standard deviation during baseline were marked as actual responses. All trials were then visually inspected to ensure that the correct maximum had been selected. Trials were rejected if there was excessive noise in the baseline or blink activity within the window 0–20 ms after startle onset. Trials with no perceptible response or with response amplitudes less than the baseline plus 3 times the standard deviation during baseline were scored as non-responses (zero) but nonetheless considered valid and included in the analyses. Startle response amplitudes were z-transformed (within participant), and responses differing more than 3 SDs from the mean were fenced in and set to ± 3 SDs. Afterwards, these scores were converted to *t* scores (*M* = 50, *SD* = 10) to standardize the scores across participants [44]. All participants had at least three valid responses per cell (maximum was 6 per Valence x Probe Interval). Notably, some participants (*n* = 10) showed non-responses on at least half of the trials. Although these non-responders are commonly excluded [43], additional analyses showed that results reported below were unaffected whether or not these participants were excluded. For simplicity, results are reported for all participants.

**EEG**. The EEG data from the 128 channels were analyzed with the FieldTrip toolbox in MATLAB [45]. The channels were visually inspected, and bad channels were excluded (for 12 participants, a mean of 3 electrodes with a range of 1 to 8 electrodes). Individual trial epochs were extracted between 200 ms before stimulus onset and 1300 ms after stimulus onset. Data were then downsampled to 250 Hz. An independent component analysis (ICA) with Runica method was used to identify eye-movement related artifacts. The topographies were inspected and those related to vertical or horizontal eye movements were removed from the data. The corrected data were visually inspected to ensure that eye movement artifacts were actually reduced, if not eliminated. Then, channels that were initially excluded were interpolated with spherical splines to obtain 128 channels for each participant. Data were referenced to the average of all 128 electrodes and for each trial, and were baseline corrected with an interval between -100 and 0 ms before stimulus onset. Individual trials were then inspected blindly in reference to their condition in a summary mode that showed the distribution of trials in terms of within trial signal variability and range. Excessive trials were excluded to minimize effects of outliers. For the ERPs of the EPN, LPP, and P3, the maximum possible number of valid trials was 24 for each unpleasant, neutral, and pleasant pictures (i.e., 8 trials per startle condition: 1500, 4500, and 7500 ms after picture onset, cf. [43]). For the EPN and LPP, the mean number of valid trials exceeded 20.6 (*SDs* < 2.6) per picture condition. For the P3, the mean number of valid trials was 19.9 (*SD* = 2.6) for pleasant pictures, 20.0 (*SD* = 2.6) for neutral pictures, and 12.6 (*SD* = 2.8) for unpleasant pictures. Notably, the difference in the number of trials for P3 does not confound the results for P3 mean amplitudes (reported below) because mean amplitudes are more robust than peak amplitudes to variations in the numbers of trials [46]. Last, event-related potentials (ERPs) were computed by averaging the trials separately for each condition.

To identify the EPN and LPP to picture onset, a difference ERP wave was computed across participants between the ERPs to picture onset for the combined pleasant and unpleasant pictures versus the ERPs to picture onset for the neutral pictures. The EPN, which is a relative negativity for arousing minus neutral pictures, was apparent between 200 and 300 ms bilaterally over occipito-temporal electrodes. Twelve electrodes (6 on each side) were used to extract mean amplitudes between 200 and 300 ms after picture onset; left: D32, A10 (PO7), A11, A12, A13, A14; and right: A26, A27, B7 (PO8), B8, B9, B10. The LPP was apparent between 400 and 800 ms over central-parietal electrodes. The following 11 electrodes were used to extract mean amplitudes between 400 and 800 ms after picture onset from electrodes from Cz down the midline to Pz: A1 (Cz), A2, A3 (CPz), A4, A19 (Pz), D1, D15, D16, C1, B1, and B2.

To identify the P3 to startle onset, a grand ERP wave was computed across participants that combined the ERPs to startle onset for all three picture types (pleasant, neutral, and unpleasant). The P3 was apparent between 300 and 380 ms over parietal electrodes. The following 3 electrodes were used to extract mean amplitudes between 300 and 380 ms after startle onset from midline electrodes around Pz: A4, A19 (Pz), and A20.

### Data analysis

Analyses focused on specific contrasts (with 1 *df*) within repeated-measures ANCOVAs to test for effects of valence (i.e., pleasant vs. unpleasant) or arousal (i.e., pleasant + unpleasant vs. neutral) in accordance with a priori hypotheses for each dependent measure. For completeness, within-subjects main effects with multiple *df*s (e.g., with 2 *df*s for emotion) are reported, and the *p* values are reported after Greenhouse-Geisser correction. For analyses of startle responses (i.e., EMG and P3), probe interval (1500 ms, 4500 ms, and 7500 ms after picture onset) was defined as an additional independent variable in the ANCOVA. Self-reported trait mindfulness was used as a continuous and centered covariate in each model, and the MAAS and the three subscales of interest on the FFMQ (Acting with awareness, FFMQ-AWA; Non-judging of inner experience, FFMQ-NJ; and Nonreactivity to inner experience, FFMQ-NR) were analyzed separately. Individual statistical tests were performed at *p* < .05, two-tailed.

Aside from treating self-reported trait mindfulness as a continuous variable in ANCOVA analyses (to maximize power in detecting effects), an alternative strategy was to perform a median split on trait mindfulness. The advantage of this approach was that the effect size of trait mindfulness (high vs. low group) as well as the effect sizes of valence and arousal could be reported as simple effect sizes (i.e., as mean differences) in the original units of each measure. Original units are often more meaningful and more easily interpreted than standardized effect sizes [47]. For each dependent measure, simple effect sizes together with confidence intervals were computed for both the primary effect (of valence or arousal) and for the group differences (based on a median split) in terms of this primary effect. In computing the group differences, scores for individuals low in mindfulness were subtracted from scores for individuals high in mindfulness; thus, negative difference scores support the hypothesis that mindfulness decreases affective reactivity.

Gender was also analyzed as a covariate and generated non-significant results for all dependent measures except for self-reported valence, *F*(1, 49) = 6.6, *p* < .013, η_*p*_^2^ = .119, with women rating unpleasant pictures as more negative than men [48]. There was no difference between men and women on any measure of trait mindfulness (*p*s > .05).

Preliminary analyses found that effects on affective reactivity were comparable for the different mindfulness scales and subscales (FFMQ-NR, FFMQ-NJ, FFMQ-AWA, and MAAS). For conciseness, detailed results are reported only for the FFMQ-NR because theoretically it is the most closely linked to affective reactivity and actually showed the strongest evidence for any moderation effects among the various subscales.

## Results

Table 1 shows means and standard deviations for the various scales and the correlations among them. Means and standard deviations for all survey measures were similar to those reported in other studies (e.g., [22, 23]), and thus, the correlations reported below between trait mindfulness and affective reactivity cannot be limited because of a restricted range in trait mindfulness [47]. Table 2 shows means and standard deviations for all measures of affective reactivity, separately for each picture category. Table 3 shows the hypotheses and inferential statistics from all contrasts regarding the relationships between FFMQ-NR and the various measures of affective reactivity. In this table, the “hypothesis” column stipulates the expected effects of valence and arousal on the different measures. For all measures, hypotheses were phrased to expect positive mean difference scores (“M_diff_”) for general effects of valence and arousal across participants. For moderating effects of FFMQ-NR, difference scores for individuals with low scores were always subtracted from difference scores for individuals with high scores. That is, if high FFMQ-NR individuals had smaller difference scores than low FFMQ-NR individuals, then the group difference (high minus low) would be negative. Because the main hypothesis for all measures was that high FFMQ-NR individuals would show lower affective reactivity than low FFMQ-NR individuals, only negative difference scores were expected.

**Table 1:** Correlations between self-reported measures.

	MAAS	FFMQ	AWA	NJ	NR	STAI-T	BDI
**FFMQ**	.68**						
**FFMQ-AWA**	.69**	.66**					
**FFMQ-NJ**	.27	.56**	.34*				
**FFMQ-NR**	.38**	.69**	.32*	.22			
**STAI-T**	- .51**	- .52**	- .53**	- .43**	- .32*		
**BDI**	- .19	- .23	- .24	- .33*	- .17	.45**	
*Mean* ^*†*^	4.1	3.5	3.6	3.8	3.1	36.5	4.6
*SD*	0.7	0.4	0.7	0.7	0.7	7.5	4.0

^†^Scores are the means across non-missing items except for STAI-T and BDI, which represent the sums across all items.

*p < .05 (2-tailed)

**p < .01 (2-tailed)

*N* = 51. MAAS = Mindful Attention Awareness Scale; FFMQ = Five Facet Mindfulness Questionnaire; FFMQ-AWA = Acting with awareness subscale; FFMQ-NJ = Non-judging subscale; FFMQ-NR = Nonreactivity subscale; STAI-T = Staite-Trait Anxiety Inventory, trait scale; BDI = Beck Depression Inventory.

**Table 2:** Means (and *SD*s) of valence and arousal measures for the three picture categories.

Dependent measure	Pleasant pictures	Neutral pictures	Unpleasant pictures
**Valence**			
Self-reported valence: Ratings (1–9)	6.6 (0.7)	5.3 (0.5)	2.6 (0.8)
Startle response: EMG (*t* scores)	48.6 (1.5)	50.4 (1.5)	50.7 (1.7)
**Arousal**			
Self-reported arousal: Ratings (1–9)	4.8 (1.3)	3.4 (1.3)	5.8 (1.4)
Sympathetic arousal: SCR (log[μS+1])	1.8 (1.1)	1.5 (0.9)	2.2 (1.5)
Early posterior negativity: EPN (μV)	3.7 (4.6)	5.5 (4.2)	4.4 (4.4)
Motivated attention to pictures: LPP (μV)	1.3 (2.3)	-0.1 (2.1)	0.8 (2.4)
Attention to startle probes: P3 (μV)	3.3 (2.7)	3.2 (2.5)	2.9 (2.4)

*N* = 51 (except for SCR, *N* = 49).

**Table 3:** Inferential statistics for valence and arousal measures across participants and as related to self-reported mindfulness (FFMQ-NR).

Affective Reactivity Measure	Hypothesis	*df*	*F*	*P*	η_*p*_^2^	M_diff_	95% *CI*	
							Lower	Upper
**Valence Effects**								
*Self-reported valence*: *Rating*								
Main effect of emotion		(2, 98)	418.2	0.001	0.895			
Valence contrast	Ple > Unp	(1, 49)	502.5	0.001	0.911	3.95	3.60	4.30
FFMQ–NR x Valence contrast	High < Low	(1, 49)	0.1	0.707	0.003	-0.10	-0.81	0.61
*Startle response*: *EMG*								
Main effect of emotion		(2, 98)	17.5	0.001	0.263			
Main effect of interval		(2, 98)	23.2	0.001	0.321			
Valence contrast	Unp > Ple	(1, 49)	26.6	0.001	0.352	2.09	1.29	2.90
FFMQ–NR x Valence contrast	High < Low	(1, 49)	0.1	0.943	0.001	-0.02	-1.65	1.61
**Arousal Effects**								
*Self-reported arousal*: *Ratings*								
Main effect of emotion		(2, 98)	86.2	0.001	0.637			
Arousal contrast	Aro > Neu	(1, 49)	140.4	0.001	0.741	1.90	1.58	2.22
FFMQ–NR x Arousal contrast	High < Low	(1, 49)	0.1	0.903	0.001	-0.09	-0.73	0.56
*Sympathetic arousal*: *SCR*								
Main effect of emotion		(2, 94)	13.6	0.001	0.225			
Arousal contrast	Aro > Neu	(1, 47)	23.2	0.001	0.330	0.54	0.31	0.77
FFMQ–NR x Arousal contrast	High < Low	(1, 47)	1.0	0.319	0.021	0.09	-0.37	0.54
*Early posterior negativity*: *EPN*								
Main effect of emotion		(2, 98)	19.2	0.001	0.282			
Arousal contrast	Neu > Aro	(1, 49)	24.6	0.001	0.334	1.43	0.84	2.01
FFMQ–NR x Arousal contrast	High < Low	(1, 49)	2.4	0.128	0.047	-0.72	-1.89	0.44
*Motivated attention to pictures*: *LPP*								
Main effect of emotion		(2, 98)	19.9	0.001	0.288			
Arousal contrast	Aro > Neu	(1, 49)	40.4	0.001	0.452	1.11	0.77	1.46
FFMQ–NR x Arousal contrast	High < Low	(1, 49)	0.1	0.812	0.001	-0.03	-0.74	0.67
*Attention to startle probes*: *P3*								
Main effect of emotion		(2, 98)	2.2	0.112	0.044			
Main effect of interval		(2, 98)	5.8	0.005	0.105			
Arousal contrast	Neu > Aro	(1, 49)	0.3	0.594	0.006	0.10	-0.27	0.46
FFMQ–NR x Arousal contrast	High < Low	(1, 49)	1.84	0.181	0.036	0.19	-0.54	0.92

*N* = 51 (except for SCR, *N* = 49). FFMQ-NR = Nonreactivity subscale of the FFMQ; Unp = unpleasant pictures; Ple = pleasant pictures; Neu = neutral pictures; Aro = unpleasant + pleasant pictures combined. Note that the inferential statistics (i.e., *df*, *F*, *p*, and *η*_p_^2^) are taken from an ANCOVA with Emotion (i.e., picture category) as within-subjects factor (and also with Interval for EMG and P3) and FFMQ-NR as a continuous and centered covariate (to maximize power). “M_diff_” refers to the mean differences between conditions or groups specified under “Hypothesis.” Accordingly, valence and arousal contrasts refer to the hypothesized differences between picture categories across participants. For all measures, these M_diff_ scores across participants were expected to be positive. To illustrate the moderating effects of FFMQ-NR, a median split was performed and difference scores for individuals with low scores (*n* = 26) were subtracted from difference scores for individuals with high scores (*n* = 25). Because for all measures, the main hypothesis was that high FFMQ-NR individuals would show lower affective reactivity than low FFMQ-NR individuals, only negative M_diff_ scores were expected for the interactions between FFMQ-NR and the valence or arousal contrasts.

As shown in Table 3 and described below, there was no evidence that mindfulness moderated affective reactivity. As shown in Table 1, however, mindfulness correlated with other self-report measures. Because it is possible that these other measures may have suppressed effects of mindfulness on affective reactivity, we conducted hierarchical multiple regression analyses for each of the affective reactivity scores (e.g., the valence effect for self-reported valence ratings, see Table 3). In a first step, gender, STAI-T, and BDI were entered. In a second step, FFMQ-NR was entered. For most measures, *R* ^2^ change < 0.5%; for EPN, *R* ^2^ change = 3.2% (*p* = .23); and for startle P3, *R* ^2^ change = 2.0% (*p* = .34). These results are consistent with the null findings reported in Table 3 and suggest that other variables did not suppress effects of mindfulness on affective reactivity.

### Valence effects

**Fig 1:**
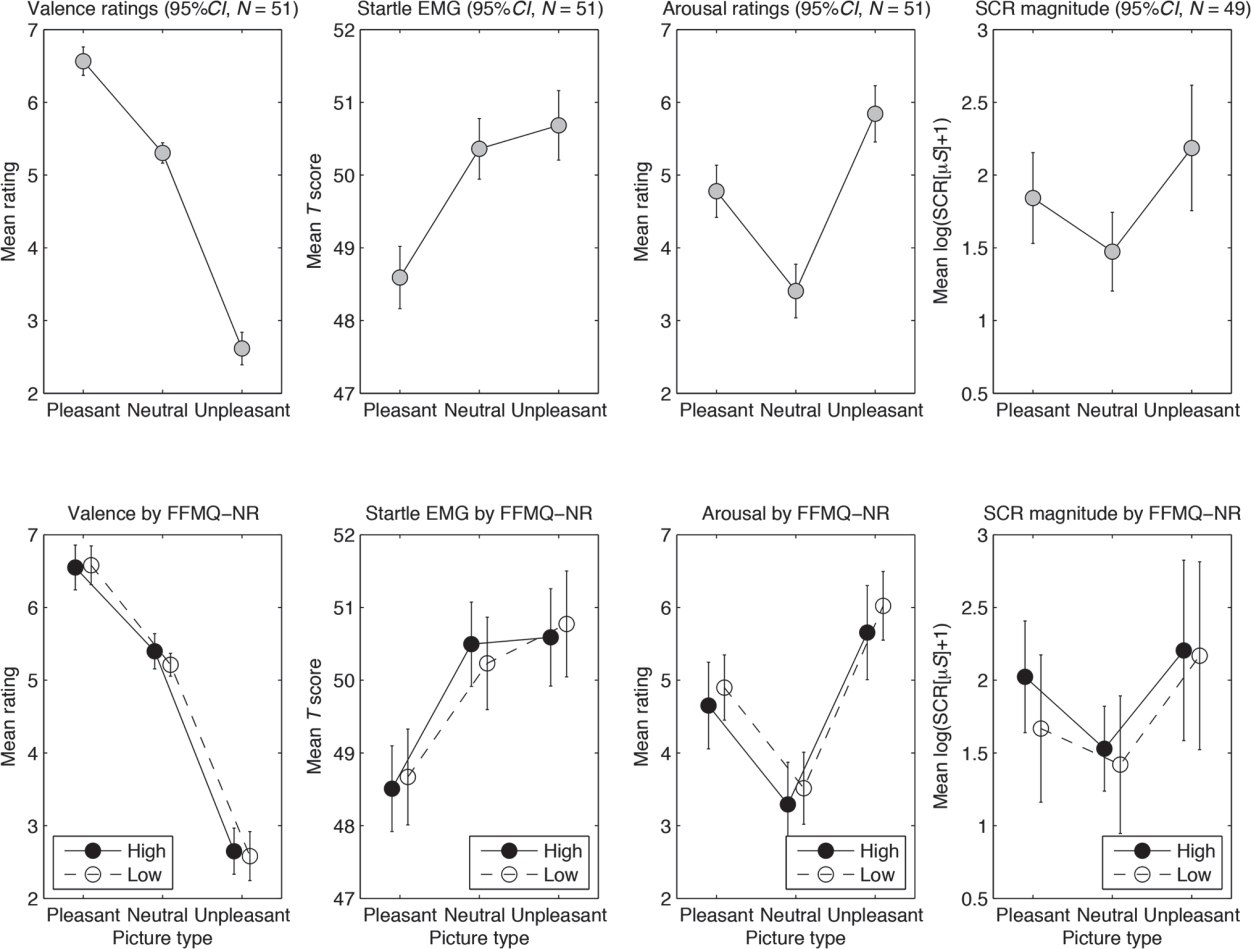
Mean responses for valence ratings, startle EMG, arousal ratings, and skin conductance responses. Mean responses are reported across picture categories for all participants (top row) and for high and low self-reported trait mindfulness separately (FFMQ-NR; bottom row). A median split was used to dichotomize high and low self-reported trait mindfulness (*N* = 51, *n* = 25 in the high group and *n* = 26 in the low group; for SCR, one individual was missing in each group). The 95% *CI* refers to each individual condition mean.

**Self-reported valence ratings.** The left-most column in Fig. 1 shows mean self-reported valence ratings for the three emotion categories across all individuals (top row) and separately for high and low FFMQ-NR individuals (bottom row). As reported in Table 3, the one-way ANCOVA showed a main effect of emotion (pleasant, neutral, unpleasant) on valence ratings. Critically, the predicted valence effect (pleasant > unpleasant) on self-reported valence ratings was found. Thus, pleasant pictures were rated as more pleasant than unpleasant pictures (with neutral pictures in between). Each of the four continuous measures of self-reported trait mindfulness was entered separately as a covariate, but neither the main effect of trait mindfulness nor the Emotion (pleasant, neutral, unpleasant) x Mindfulness interaction was statistically significant for any of the measures.

**Affect-modulated startle eyeblink response (EMG)**. The second column in Fig. 1 shows mean startle eyeblink responses for the three emotion categories across all individuals (top row) and separately for high and low FFMQ-NR individuals (bottom row). As reported in Table 3, the two-factorial ANCOVA (with emotion and interval) on startle EMG showed a main effect of emotion and also the predicted valence effect. Thus, the startle response was greater for unpleasant than pleasant pictures (with neutral pictures in between). There was also a main effect of probe interval, showing that startle amplitudes generally increased with longer probe intervals (mean *t* scores were 48.5 at 1500 ms, 49.7 at 4500 ms, and 51.4 at 7500 ms). This replicates the common finding that startle eyeblink response increases with interval [43]. There were no statistically significant main effects of trait mindfulness or Emotion x Mindfulness interactions.

### Arousal effects

**Self-reported arousal ratings**. The third column in Fig. 1 shows mean self-reported arousal ratings for the three emotion categories across all individuals (top row) and separately for high and low FFMQ-NR individuals (bottom row). As reported in Table 3, the one-way ANCOVA showed a main effect of emotion on arousal ratings. The predicted arousal effect (pleasant + unpleasant minus neutral) was also found, with pleasant and unpleasant pictures being rated as more arousing than neutral pictures. There were no statistically significant main effects of trait mindfulness or Emotion x Mindfulness interactions.

**Sympathetic arousal (SCR)**. The fourth column in Fig. 1 shows the mean SCR magnitude for the three emotion categories across all individuals (top row) and separately for high and low FFMR-NR individuals (bottom row). As reported in Table 3, the one-way ANCOVA showed a main effect of emotion on the SCR magnitude as well as the expected arousal effect. As predicted, arousing (i.e., pleasant and unpleasant) pictures elicited greater responses than neutral pictures. There were no statistically significant main effects of trait mindfulness or Emotion x Mindfulness interactions.

**Fig 2:**
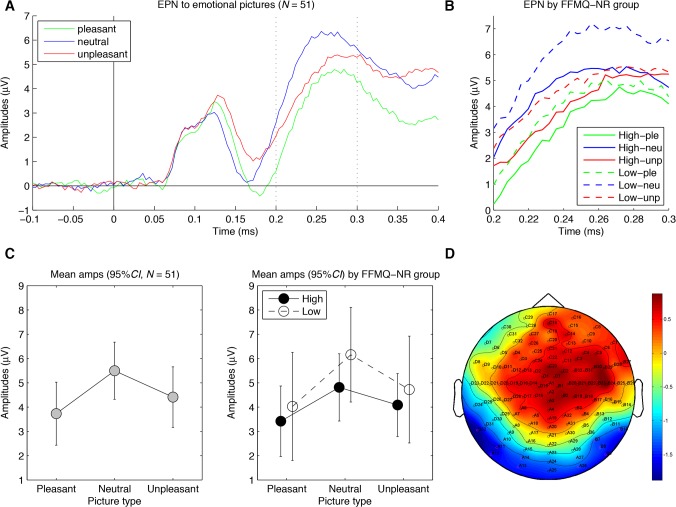
Results for the early posterior negativity (EPN) to picture onset. (A) Grand average waveforms of the EPN-relevant electrodes for the three picture categories across participants. Note that the EPN per se is a relative negativity between 200 and 300 ms after picture onset for pleasant and unpleasant pictures versus neutral pictures. (B) Magnified version of (A) for individuals with high and low self-reported trait mindfulness separately. (C) Graphs depicting the mean amplitudes of the EPN-relevant electrodes between 200 and 300 ms after picture onset for the three picture categories for all participants (left, *N* = 51) and for high (*n* = 25) and low (*n* = 26) self-reported trait mindfulness separately (right). (D) Topographical distribution of the mean amplitude differences between 200 and 300 ms after picture onset between the combined pleasant and unpleasant pictures versus neutral pictures, with electrodes selected for analysis in magenta (left: D32, A10 (PO7), A11, A12, A13, A14; and right: A26, A27, B7 (PO8), B8, B9, B10). The 95% *CI* refers to each individual condition mean. FFMQ-NR = Nonreactivity subscale of the FFMQ.

**Early posterior negativity (EPN).** In Fig. 2, the mean EPN-relevant amplitudes are shown for the three emotion categories across all individuals (A and C) and separately for high and low FFMQ-NR individuals (B and D). As reported in Table 3, the one-way ANCOVA showed both a main effect of emotion on EPN-relevant amplitudes and the expected arousal effect. As predicted, EPN-relevant amplitudes to pleasant and unpleasant pictures were less positive (i.e., relatively negative) than amplitudes to neutral pictures. Note, however, that the mean difference score across participants in Table 3 was expressed as neutral minus arousing pictures to yield positive values for effects of arousal, as for the other measures. Neither the main effects of trait mindfulness nor the Emotion x Mindfulness interactions were statistically significant.

**Fig 3:**
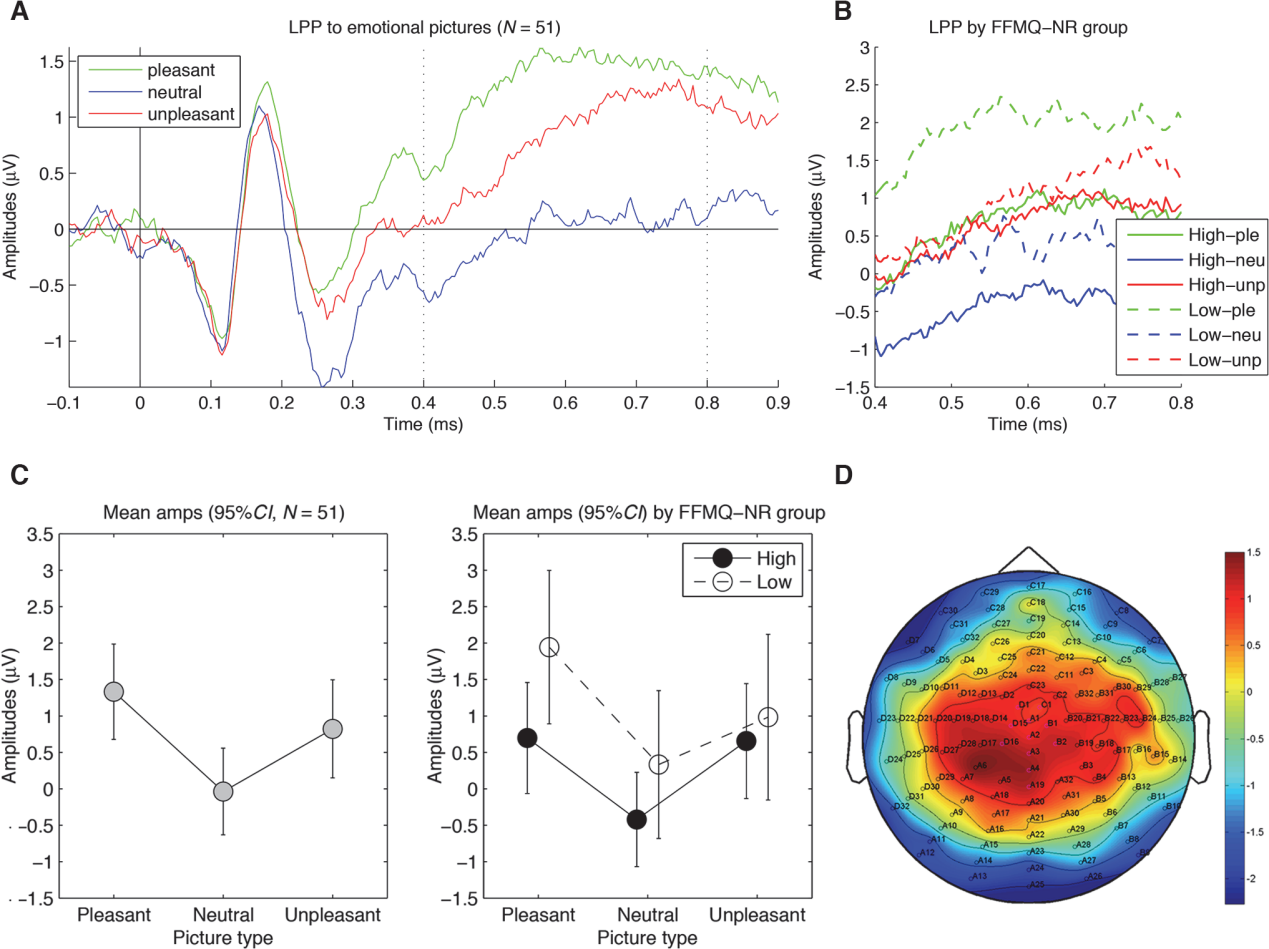
Results for the late positive potential (LPP) to picture onset. (A) Grand average waveforms of the LPP-relevant electrodes for the three picture categories across participants. Note that the arousal effect on the LPP is apparent as the relative positivity between 400 and 800 ms after picture onset for arousing (i.e., pleasant and unpleasant) pictures versus neutral pictures. (B) Magnified version of (A) for individuals with high and low self-reported trait mindfulness separately. (C) Graphs depicting the mean amplitudes of the LPP-relevant electrodes between 400 and 800 ms after picture onset for the three picture categories for all participants (left, *N* = 51) and for high (*n* = 25) and low (*n* = 26) self-reported trait mindfulness separately (right). (D) Topographical distribution of the mean amplitude differences between 400 and 800 ms after picture onset between the arousing (i.e., combined pleasant and unpleasant) pictures versus neutral pictures, with electrodes selected for analysis in magenta (A1 (Cz), A2, A3 (CPz), A4, A19 (Pz), D1, D15, D16, C1, B1, and B2). The 95% *CI* refers to each individual condition mean. FFMQ-NR = Nonreactivity subscale of the FFMQ.

**Motivated attention to pictures (LPP)**. In Fig. 3, the mean LPP amplitudes are shown for the three emotion categories across all individuals (A and C) and separately for high and low FFMQ-NR individuals (B and D). As reported in Table 3, the one-way ANCOVA revealed a main effect of emotion on LPP amplitudes and the expected arousal effect. As predicted, LPP amplitudes to pleasant and unpleasant pictures were greater than to neutral pictures. There were no statistically significant main effects of trait mindfulness or Emotion x Mindfulness interactions.

**Fig 4:**
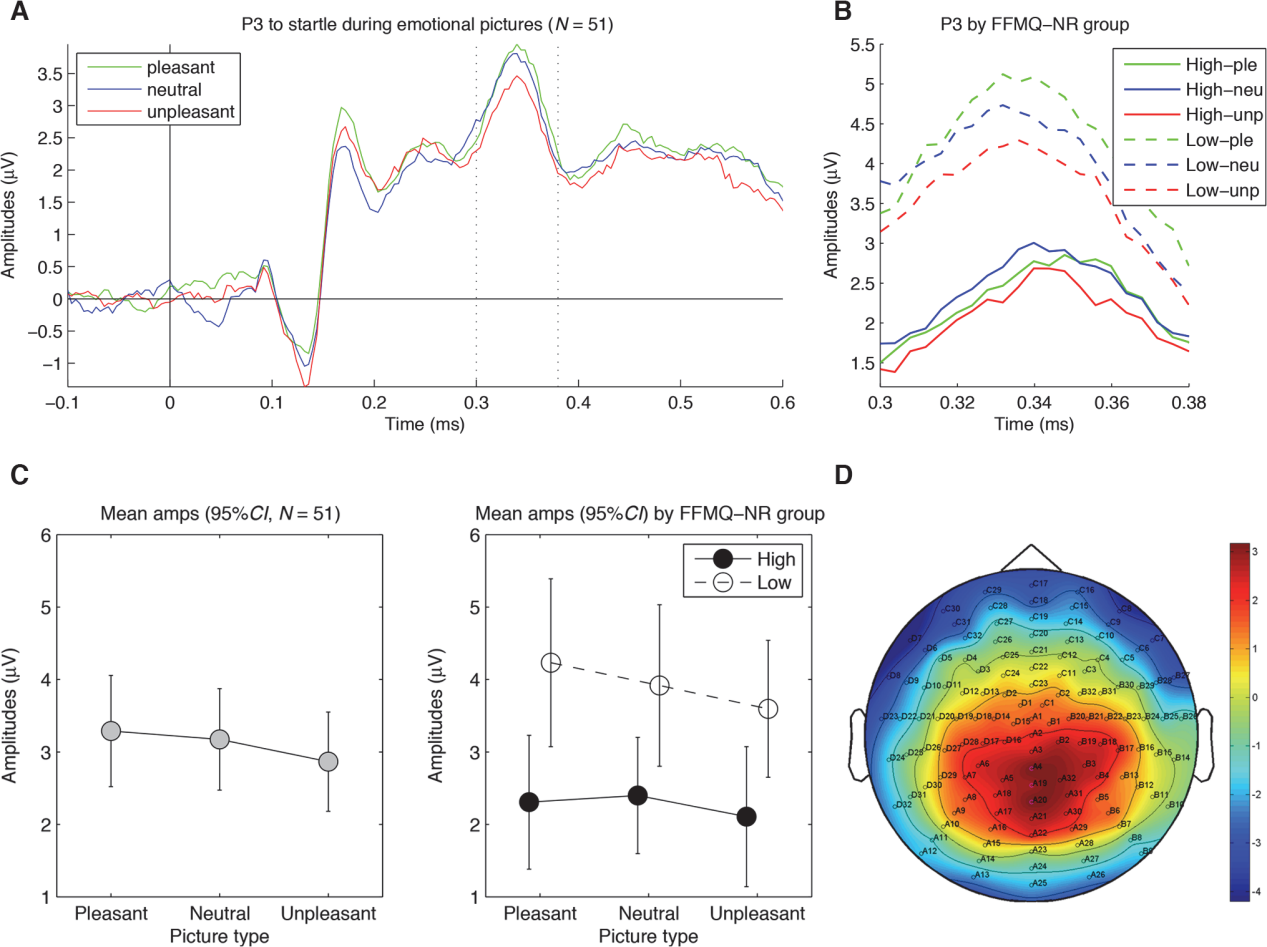
Results for the P3 to startle probes. (A) Grand average waveforms of the P3-relevant electrodes for the three picture categories across participants. Note that the P3 per se is the positivity between 300 and 380 ms after startle onset, and an arousal effect on the P3 would be apparent as a greater positivity for neutral pictures than arousing (i.e., pleasant and unpleasant) pictures. (B) Magnified version of (A) for individuals with high and low self-reported trait mindfulness separately. (C) Graphs depicting the mean amplitudes of the P3-relevant electrodes between 300 and 380 ms after picture onset for the three picture categories for all participants (left, *N* = 51) and for high (*n* = 25) and low (*n* = 26) self-reported trait mindfulness separately (right). (D) Topographical distribution of the mean amplitude differences between 300 and 380 ms after picture onset between the combined pleasant and unpleasant pictures versus neutral pictures, with electrodes selected for analysis in magenta (A4, A19 (Pz), and A20). The 95% *CI* refers to each individual condition mean. FFMQ-NR = Nonreactivity subscale of the FFMQ.

**Attention to startle probes (P3)**. In Fig. 4, the mean P3 amplitudes are shown for the three emotion categories across all individuals (A and C) and separately for high and low FFMQ-NR individuals (B and D). As reported in Table 3, the two-factorial ANCOVA showed no main effect of emotion, and the hypothesized arousal effect (neutral > arousing pictures) was not statistically significant. Notably, a main effect of probe interval showed that P3 amplitudes increased with longer probe intervals (mean P3 amplitudes were 2.7 μV at 1500 ms, 3.1 μV at 4500 ms, and 3.5 μV at 7500 ms), consistent with previous findings [37]. Additionally, there was a main effect of trait mindfulness (FFMQ-NR) on P3 amplitudes, *F*(2, 98) = 4.8, *p* = .033, η_*p*_^2^ = .089, suggesting that P3 amplitudes were generally decreased with higher mindfulness. However, none of the Emotion x Mindfulness interactions was statistically significant.

## Discussion

The purpose of the present study was to investigate the degree to which self-reported trait mindfulness is related to affective reactivity during passive viewing of emotional pictures. We employed multiple measures of affective reactivity to capture effects of valence (using self-reported valence ratings and startle eyeblink responses) and effects of arousal (using self-reported arousal ratings, skin conductance responses, EPN amplitudes to pictures, LPP amplitudes to pictures, and P3 amplitudes to startle probes). Across individuals, typical effects of valence and arousal were found for all dependent measures except for P3 amplitudes to startle probes. Despite these typical effects of picture valence and arousal, various aspects of self-reported trait mindfulness did not generally moderate these effects.

Importantly, across all individuals, the hypothesized valence and arousal effects were substantial for all dependent measures (except for P3 amplitudes to startle probes; see Table 3). For example, participants rated their subjective valence to pleasant pictures as nearly 4 points higher than to unpleasant pictures (on a 9-point scale; 95% *CI*: 3.60 to 4.30 points), and their subjective arousal to arousing (pleasant + unpleasant) pictures as nearly 2 points higher than to neutral pictures (95% *CI*: 1.58 to 2.22 points). These results indicated successful manipulation of emotion in terms of valence and arousal and thus warranted further examination of the moderating effects of self-reported trait mindfulness.

For all successfully manipulated dependent measures, results suggested no moderation effects from self-reported trait mindfulness and negligible effect sizes. This was true for all measured aspects of self-reported trait mindfulness (i.e., FFMQ-AWA, FFMQ-NJ, FFMQ-NR, and MAAS), but as stated earlier, we focused on FFMQ-NR to illustrate these effects. Aside from treating self-reported trait mindfulness as a continuous variable in ANCOVA analyses (to maximize power in detecting effects), an alternative strategy used a median split to dichotomize the trait mindfulness. The advantage of this procedure was that it permitted assessment of trait mindfulness effects in terms of simple effect sizes, which are expressed on the original units of analysis and tend to be easier to interpret than standardized effect sizes [47]. As shown in Table 3, the point estimates of the effect sizes of trait mindfulness suggested rather negligible effect sizes (i.e., effect sizes were close to zero). In addition, the interval estimates of these effect sizes, as indicated by the 95% confidence intervals, were arguably quite narrow. For example, the valence effect (i.e., pleasant minus unpleasant pictures) on valence ratings yielded a mean difference of about 4 points on a 9-point scale, but the moderating effect of group on this valence effect was only -0.10, 95% CI [-0.81, 0.61]. Thus, our best guess from these data is that the mean group difference in the population is -0.10 on the 9-point valence scale (although a mean difference of -0.81 or even +0.61 cannot be dismissed). Arguably, such a group difference may be negligible.

If the present findings are taken at face value, they imply that self-reported trait mindfulness is, on the whole, unrelated to affective reactivity while viewing emotional pictures. As reviewed above, our findings fit in with the rather limited and mixed evidence from studies operationalizing mindfulness as a self-reported trait [19–21]. In addition, our findings are consistent with other theoretical and empirical data suggesting that trait mindfulness is problematic both as a self-reported measure and as a construct.

Specifically, there has been much debate about whether the self-reported trait mindfulness scales, such as the MAAS and FFMQ, are actually measuring mindfulness [12]. The MAAS, for example, consists of 15 items that are all reversed scored, and each measures the perceived frequency of inattention or “mindlessness.” While the opposite of perceived inattention may arguably be related to mindfulness, it clearly does not cover other aspects of mindfulness as it is generally conceptualized in the psychological literature, and recent item response theory analyses have supported this idea [49]. The FFMQ suffers from the same problem as the MAAS on the awareness subscale (FFMQ-AWA), and it has also been found to be differentially interpreted by meditators and non-meditators [50]. In addition, it has been suggested that a certain level of mindfulness is required for people to report mindfulness accurately. Accordingly, if individuals are not aware of their inattention or reactivity, they are unlikely to report it accurately [12]. This is problematic as inattentive or reactive individuals may incorrectly report high levels of awareness or non-reactivity.

Further, it has been suggested that the entire construct of trait mindfulness is problematic. Grossman and Van Dam [12] cite Buddhist texts to suggest that mindfulness is better characterized as a state or process, rather than a trait. If this is indeed the case, mindfulness may not be a stable quality that can be assessed as a trait variable and therefore may not relate to affective reactivity, which is proposed to reflect stable individual differences [51].

In line with this, authors such as Desbordes and colleagues [9] have suggested moving beyond mindfulness to focus on the outcomes of mindfulness practice, such as the development of equanimity. The authors define equanimity as “…an even-minded mental state or dispositional tendency toward all experiences or objects, regardless of their affective valence… or source” (p. 2) and propose that this may be a more useful construct than trait mindfulness. Equanimity is characterized as a stable quality developed through mindfulness practice, and therefore may be a more fruitful trait-level outcome variable. Because self-reported scales of equanimity are available (e.g., Non-attachment Scale) [52] and because equanimity has been theorized to produce physiological changes in reactivity (e.g., skin conductance responses and heart rate variability) [9], the present design lends itself to investigate whether equanimity decreases affective reactivity, and whether the development of equanimity mediates the relationship between mindfulness practice and affective reactivity.

In closing, we note several limitations of the present study. First, for startle P3, no emotion effects across subjects were obtained. This null finding is puzzling. The present task showed clear emotion effects of the pictures across subjects on all the other measures (i.e., self-report, SCR, EPN, LPP, and startle EMG), providing a convincing manipulation check of emotion. Indeed, the startle EMG, which was recorded to the same startle sounds as the startle P3, showed clear emotion effects. This rules out the possibility that the startle probes were ineffective. Further, Fig. 4 shows that our recording of the startle P3 showed the typical pattern of a positive wave peaking between 300 and 400 ms over central-parietal midline [35, 36], as well as a general increase with probe interval after picture onset [37]. Thus, our results across subjects showed the canonical startle P3 irrespective of picture category, but this response was not moderated by emotion. Because the upper limit of the 95% CI for the effect of arousal on startle P3 was quite small (< 0.5 μV, see Table 3), our findings suggest that for the present study design, emotion effects on the startle P3 may not be readily apparent. However, the limits of the 95% CI for the moderating effects of trait mindfulness suggested a possible group difference of about 1 μV (see Table 3). This means that although emotion effects may not be apparent across subjects, there may be group differences for emotion effects. However, any conclusions about effects of trait mindfulness on startle P3 should be drawn cautiously given the null finding of emotion effects on startle P3 across subjects.

Second, our findings are limited to mindfulness in terms of a self-reported trait. As discussed above, mindfulness may be more meaningful when considered as a state or process [12]. Further, equanimity may be a more useful trait-level concept than trait mindfulness [9]. Critically, because the present findings are limited to self-reported trait mindfulness, they are not in conflict with many studies that show clear trait effects of meditation practice [53].

Third, although our findings do not support a role of self-reported trait mindfulness in affective reactivity to emotional pictures, only a future meta-analysis that combines many studies with numerous individuals may accurately assess the degree to which self-reported trait mindfulness affects affective reactivity. Importantly, our results provide simple and standardized effect sizes to facilitate a future meta analysis [54].

Fourth, the present results were obtained with emotional pictures and do not necessarily generalize to other emotional stimuli. For example, self-reported trait mindfulness has been shown to reduce cortisol and negative affect to social evaluative stress [55], to reduce heart rate and subjective distress to CO_2_ inhalation while engaging in suppression [56], to dampen neuroaffective reactions (i.e., the feedback-related negativity) to rewarding stimuli during a time-estimation task [57], and to decrease cortisol and negative affect and to increase positive cognitive appraisals during a conflict discussion in heterosexual couples [58]. These results indicate that the present results with emotional pictures may not generalize to other emotional stimuli.

Fifth, participants in the present study viewed emotional and neutral pictures passively and were not instructed to view these pictures in a particular (i.e., mindful) way. Passive picture viewing may be an ideal way to capture the uninstructed and spontaneous effects of self-reported trait mindfulness and thus resembles studies on the uninstructed use of emotion regulation strategies. For example, in a study by Drabant et al. [59], participants completed the Emotion Regulation Questionnaire to assess their use of reappraisal strategies in everyday life. They then performed an identity matching task with negative faces (or shapes as control), and the emotional expressions of the faces were task irrelevant. Even though participants did not receive any specific task instructions to use reappraisal during the task, the fMRI results resembled those obtained with studies in which participants were explicitly instructed to use reappraisal. That is, the self-reported use of reappraisal strategies correlated positively with prefrontal activation and negatively with amygdala activity to the emotional faces. In contrast to these findings, however, the present null findings suggest two possibilities. Either self-reported trait mindfulness does not result in the spontaneous use of a mindful strategy while viewing emotional pictures, or alternatively, self-reported trait mindfulness does result in the spontaneous use of a mindful strategy, but this strategy is ineffective in changing affective reactivity to emotional pictures.

Critically, because our findings were obtained with passive viewing, they do not necessarily generalize to findings with active tasks that either instruct subjects to adopt a particular task strategy (e.g., reappraisal) or induce a mindful task strategy implicitly because of the nature of the task. In these cases, self-reported trait mindfulness correlates with affective reactivity. For example, Modinos et al. [60] instructed participants to either reappraise negative IAPS pictures or to attend to them. Individual differences in self-reported trait mindfulness correlated positively with prefrontal activation during reappraisal (vs. attending), and the prefrontal activation correlated negatively with amygdala activation. In a study by Creswell et al. [61], participants were shown negative faces and had to match either the emotional expression with labels (e.g., angry or scared) or the gender of the face with gender-typical names (e.g., Samuel or Helen). Results showed that individual differences in self-reported trait mindfulness correlated positively with increased prefrontal activation and negatively with amygdala activation during the affect labeling task (vs. the gender labeling task). The authors argued that affect labeling corresponds to a mindful perspective. When individuals with high self-reported trait mindfulness performed the affect labeling task (and were thus, mindful), they showed reduced affective reactivity. Taken together, these results suggest that self-reported trait mindfulness moderates affective reactivity to emotional pictures during active tasks with specific instructions. Therefore, future research should employ similar measures as used here to determine whether or not self-reported trait mindfulness predicts affective reactivity to emotional pictures when a mindfulness strategy is evoked during the task.

## Conclusion

The present study employed a motivational model of emotion with dimensions of valence and arousal, and recorded multiple emotion measures to examine the degree to which self-reported trait mindfulness moderates affective reactivity to emotional pictures during passive picture viewing. Although most dependent measures showed clear effects of emotion, different aspects of self-reported trait mindfulness, on the whole, did not moderate these effects. The point estimates of effect sizes were close to zero and the interval estimates were arguably also quite narrow, suggesting negligible effects. The absence of an effect of self-reported trait mindfulness on affective reactivity is consistent with arguments that current questionnaires measuring trait mindfulness may not be valid measures of mindfulness or that self-reported trait mindfulness per se may not be related to spontaneous affective reactivity to emotional pictures.

## Supporting Information

S1 Table IAPS picture codes. (DOCX)

S1 Dataset Zip file containing the data in SPSS format (*.sav) and as a Tab-delimited file (*.dat) together with a summary of variable and value labels. (ZIP)

## References

[1] Chambers R, Gullone E, Allen NB. Mindful emotion regulation: An integrative review. Clin Psychol Rev. 2009;29(6):560–72. 10.1016/j.cpr.2009.06.005 PubMed PMID: . 19632752

[2] Kabat-Zinn J. An outpatient program in behavioral medicine for chronic pain patients based on the practice of mindfulness meditation—theoretical considerations and preliminary-results. Gen Hosp Psychiatry. 1982;4(1):33–47. 10.1016/0163-8343(82)90026-3 PubMed PMID: . 7042457

[3] Keng SL, Smoski MJ, Robins CJ. Effects of mindfulness on psychological health: A review of empirical studies. Clin Psychol Rev. 2011;31(6):1041–56. 10.1016/j.cpr.2011.04.006 PubMed PMID: . 21802619 PMC3679190

[4] Jain S, Shapiro SL, Swanick S, Roesch SC, Mills PJ, Bell I, et al A randomized controlled trial of mindfulness meditation versus relaxation training: Effects on distress, positive states of mind, rumination, and distraction. Ann Behav Med. 2007;33(1):11–21. 10.1207/s15324796abm3301_2 PubMed PMID: . 17291166

[5] Farb NAS, Segal ZV, Mayberg H, Bean J, McKeon D, Fatima Z, et al Attending to the present: mindfulness meditation reveals distinct neural modes of self-reference. Soc Cogn Affect Neurosci. 2007;2(4):313–22. 10.1093/scan/nsm030 PubMed PMID: . 18985137 PMC2566754

[6] Lykins ELB, Baer RA. Psychological functioning in a sample of long-term practitioners of mindfulness meditation. J Cogn Psychother. 2009;23(3):226–41. 10.1891/0889-8391.23.3.226

[7] Feldman G, Hayes A, Kumar S, Greeson J, Laurenceau JP. Mindfulness and emotion regulation: The development and initial validation of the Cognitive and Affective Mindfulness Scale-Revised (CAMS-R). J Psychopathol Behav Assess. 2007;29(3):177–90. 10.1007/s10862-006-9035-8 PubMed PMID: .

[8] Roemer L, Lee JK, Salters-Pedneault K, Erisman SM, Orsillo SM, Mennin DS. Mindfulness and emotion regulation difficulties in generalized anxiety disorder: Preliminary evidence for independent and overlapping contributions. Behav Ther. 2009;40(2):142–54. 10.1016/j.beth.2008.04.001 PubMed PMID: . 19433145 PMC3719394

[9] Desbordes G, Gard T, Hoge EA, Hölzel BK, Kerr C, Lazar SW, et al. Moving beyond mindfulness: Defining equanimity as an outcome measure in meditation and contemplative research. Mindfulness. 2014:1–17. doi: 10.1007/s12671-013-0269-8 PMC4350240 25750687

[10] Davidson RJ. Affective neuroscience and psychophysiology: Toward a synthesis. Psychophysiology. 2003;40(5):655–65. 10.1111/1469-8986.00067 PubMed PMID: . 14696720

[11] Nolen-Hoeksema S. The role of rumination in depressive disorders and mixed anxiety/depressive symptoms. J Abnorm Psychol. 2000;109(3):504–11. 10.1037/0021-843x.109.3.504 PubMed PMID: . 11016119

[12] Grossman P, Van Dam NT. Mindfulness, by any other name…: trials and tribulations of Sati in Western psychology and science. Contemporary Buddhism. 2011;12(1):219–39. 10.1080/14639947.2011.564841 PubMed PMID: .

[13] Kabat-Zinn J. Wherever you go there you are: Mindfulness meditation in everyday life New York: Hyperion; 1994.

[14] Bishop SR, Lau M, Shapiro S, Carlson L, Anderson ND, Carmody J, et al Mindfulness: A proposed operational definition. Clin Psychol. 2004;11(3):230–41. 10.1093/clipsy/bph077 PubMed PMID: .

[15] Hölzel BK, Lazar SW, Gard T, Schuman-Olivier Z, Vago DR, Ott U. How does mindfulness meditation work? Proposing mechanisms of action from a conceptual and neural perspective. Perspectives on Psychological Science. 2011;6(6):537–59. 10.1177/1745691611419671 PubMed PMID: . 26168376

[16] Arch JJ, Craske MG. Mechanisms of mindfulness: Emotion regulation following a focused breathing induction. Behav Res Ther. 2006;44(12):1849–58. 10.1016/j.brat.2005.12.007 PubMed PMID: . 16460668

[17] Ortner CNM, Kilner SJ, Zelazo PD. Mindfulness meditation and reduced emotional interference on a cognitive task. Motiv Emot. 2007;31(4):271–83. 10.1007/s11031-007-9076-7 PubMed PMID: .

[18] Taylor VA, Grant J, Daneault V, Scavone G, Breton E, Roffe-Vidal S, et al Impact of mindfulness on the neural responses to emotional pictures in experienced and beginner meditators. Neuroimage. 2011;57(4):1524–33. 10.1016/j.neuroimage.2010.06.001 PubMed PMID: . 21679770

[19] Ostafin BD, Brooks JJ, Laitem M. Affective reactivity mediates an inverse relation between mindfulness and anxiety. Mindfulness. 2013:1–9. doi: 10.1007/s12671-013-0206-x.

[20] Sauer S, Walach H, Schmidt S, Hinterberger T, Horan M, Kohls N. Implicit and explicit emotional behavior and mindfulness. Conscious Cogn. 2011;20(4):1558–69. 10.1016/j.concog.2011.08.002 PubMed PMID: . 21885296

[21] Brown KW, Goodman RJ, Inzlicht M. Dispositional mindfulness and the attenuation of neural responses to emotional stimuli. Soc Cogn Affect Neurosci. 2013;8(1):93–9. 10.1093/scan/nss004 PubMed PMID: . 22253259 PMC3541486

[22] Baer RA, Smith GT, Hopkins J, Krietemeyer J, Toney L. Using self-report assessment methods to explore facets of mindfulness. Assessment. 2006;13(1):27–45. 10.1177/1073191105283504 PubMed PMID: . 16443717

[23] Brown KW, Ryan RM. The benefits of being present: Mindfulness and its role in psychological well-being. J Pers Soc Psychol. 2003;84(4):822–48. 10.1037/0022-3514.84.4.822 PubMed PMID: . 12703651

[24] Gootjes L, Franken IHA, Van Strien JW. Cognitive emotion regulation in yogic meditative practitioners sustained modulation of electrical brain potentials. J Psychophysiol. 2011;25(2):87–94. 10.1027/0269-8803/a000043 PubMed PMID: .

[25] Sobolewski A, Holt E, Kublik E, Wrobel A. Impact of meditation on emotional processing-A visual ERP study. Neurosci Res. 2011;71(1):44–8. 10.1016/j.neures.2011.06.002 PubMed PMID: . 21689695

[26] Olofsson JK, Nordin S, Sequeira H, Polich J. Affective picture processing: An integrative review of ERP findings. Biol Psychol. 2008;77(3):247–65. 10.1016/j.biopsycho.2007.11.006 PubMed PMID: . 18164800 PMC2443061

[27] Lang PJ, Bradley MM. Emotion and the motivational brain. Biol Psychol. 2010;84(3):437–50. 10.1016/j.biopsycho.2009.10.007 PubMed PMID: . 19879918 PMC3612949

[28] Koch M. The neurobiology of startle. Prog Neurobiol. 1999;59(2):107–28. 10.1016/s0301-0082(98)00098-7 PubMed PMID: . 10463792

[29] Blumenthal TD, Cuthbert BN, Filion DL, Hackley S, Lipp OV, van Boxtel A. Committee report: Guidelines for human startle eyeblink electromyographic studies. Psychophysiology. 2005;42(1):1–15. 10.1111/j.1469-8986.2005.00271.x 15720576

[30] Dawson ME, Schell AM, Filion DL. The electrodermal system In: Cacioppo JT, Tassinary LG, Berntson G, editors. Handbook of Psychophysiology. 3rd ed. New York: Cambridge University Press; 2007 p. 159–81.

[31] Lang PJ, Greenwald MK, Bradley MM, Hamm AO. Looking at pictures: Affective, facial, visceral, and behavioral reactions. Psychophysiology. 1993;30(3):261–73. PubMed PMID: . 8497555 10.1111/j.1469-8986.1993.tb03352.x

[32] Schupp HT, Flaisch T, Stockburger J, Junghöfer M. Emotion and attention: Event-related brain potential studies In: Anders S, Ende G, Junghöfer M, Kissler J, Wildgruber D, editors. Progress in Brain Research: Understanding Emotions. 156: Elsevier: Amsterdam; 2006 p. 31–51. 17015073 10.1016/S0079-6123(06)56002-9

[33] Wiens S, Syrjänen E. Directed attention reduces processing of emotional distracters irrespective of valence and arousal level. Biol Psychol. 2013;94(1):44–54. 10.1016/j.biopsycho.2013.05.001 PubMed PMID: . 23669534

[34] Schupp HT, Cuthbert BN, Bradley MM, Cacioppo JT, Ito T, Lang PJ. Affective picture processing: The late positive potential is modulated by motivational relevance. Psychophysiology. 2000;37(2):257–61. 10731776

[35] Schupp HT, Cuthbert BN, Bradley MM, Birbaumer N, Lang PJ. Probe P3 and blinks: Two measures of affective startle modulation. Psychophysiology. 1997;34:1–6. 9009802 10.1111/j.1469-8986.1997.tb02409.x

[36] Keil A, Bradley MM, Junghöfer M, Russmann T, Lowenthal W, Lang PJ. Cross-modal attention capture by affective stimuli: Evidence from event-related potentials. Cogn Affect Behav Neurosci. 2007;7(1):18–24. PubMed PMID: . 17598731 10.3758/cabn.7.1.18

[37] Bradley MM, Codispoti M, Lang PJ. A multi-process account of startle modulation during affective perception. Psychophysiology. 2006;43(5):486–97. 10.1111/j.1469-8986.2006.00412.x 16965611

[38] Beck AT, Steer RA, Garbin MG. Psychometric properties of the beck depression inventory—25 years of evaluation. Clin Psychol Rev. 1988;8(1):77–100. 10.1016/0272-7358(88)90050-5 PubMed PMID: .

[39] Spielberger CD. Manual for the State-Trait Anxiety Inventory Palo Alto, CA: Consulting Psychologists; 1983.

[40] Lilja JL, Frodi-Lundgren A, Hanse JJ, Josefsson T, Lundh L-G, Sköld C, et al Five Facets Mindfulness Questionnaire—Reliability and factor structure: A Swedish version. Cogn Behav Ther. 2011;40(4):291–303. 10.1080/16506073.2011.580367 21770845

[41] Lang PJ, Bradley MM, Cuthbert BN. International Affective Picture System (IAPS): Affective ratings of pictures and instruction manual Technical report a-8. Gainesville, FL: University of Florida; 2008.

[42] Bradley MM, Lang PJ. Measuring emotion—the Self-Assessment Mannequin and the semantic differential. J Behav Ther Exp Psychiatry. 1994;25(1):49–59. PubMed PMID: . 7962581 10.1016/0005-7916(94)90063-9

[43] Larson CL, Ruffalo D, Nietert JY, Davidson RJ. Stability of emotion-modulated startle during short and long picture presentation. Psychophysiology. 2005;42(5):604–10. 10.1111/j.1469-8986.2005.00345.x PubMed PMID: . 16176383

[44] Bradley MM, Codispoti M, Cuthbert BN, Lang PJ. Emotion and motivation I: Defensive and appetitive reactions in picture processing. Emotion. 2001;1(3):276–98. 12934687

[45] Oostenveld R, Fries P, Maris E, Schoffelen JM. FieldTrip: Open source software for advanced analysis of MEG, EEG, and invasive electrophysiological data. Comput Intell Neurosci. 2011;2011:156869 10.1155/2011/156869 PubMed PMID: 21253357 PMC3021840

[46] Luck SJ. An introduction to the event-related potential technique Cambridge, MA: MIT Press; 2005.

[47] Baguley T. Standardized or simple effect size: What should be reported? Br J Psychol. 2009;100:603–17. 10.1348/000712608x377117 PubMed PMID: . 19017432

[48] Syrjänen E, Wiens S. Gender moderates valence effects on the late positive potential to emotional distracters. Neurosci Lett. 2013;551(0):89–93. 10.1016/j.neulet.2013.07.018 23886486

[49] Van Dam NT, Earleywine M, Borders A. Measuring mindfulness? An item response theory analysis of the mindful attention awareness scale. Pers Individ Dif. 2010;49(7):805–10. 10.1016/j.paid.2010.07.020 PubMed PMID: .

[50] Van Dam NT, Earleywine M, Danoff-Burg S. Differential item function across meditators and non-meditators on the Five Facet Mindfulness Questionnaire. Pers Individ Dif. 2009;47(5):516–21. 10.1016/j.paid.2009.05.005 PubMed PMID: .

[51] Davidson RJ. Affective style, psychopathology, and resilience: Brain mechanisms and plasticity. Am Psychol. 2000;55(11):1196–214. PubMed PMID: . 11280935 10.1037//0003-066x.55.11.1196

[52] Sahdra BK, Shaver PR, Brown KW. A scale to measure nonattachment: A buddhist complement to western research on attachment and adaptive functioning. J Pers Assess. 2010;92(2):116–27. 10.1080/00223890903425960 PubMed PMID: . 20155561

[53] Cahn BR, Polich J. Meditation states and traits: EEG, ERP, and neuroimaging studies. Psychol Bull. 2006;132(2):180–211. 10.1037/0033-2909.132.2.180 PubMed PMID: . 16536641

[54] Cumming G. The New Statistics: Why and how. Psychol Sci. 2014;25(1):7–29. 10.1177/0956797613504966 PubMed PMID: . 24220629

[55] Brown KW, Weinstein N, Creswell JD. Trait mindfulness modulates neuroendocrine and affective responses to social evaluative threat. Psychoneuroendocrinology. 2012;37(12):2037–41. 10.1016/j.psyneuen.2012.04.003 PubMed PMID: . 22626868 PMC5087919

[56] Bullis JR, Boe HJ, Asnaani A, Hofmann SG. The benefits of being mindful: Trait mindfulness predicts less stress reactivity to suppression. J Behav Ther Exp Psychiatry. 2014;45(1):57–66. 10.1016/j.jbtep.2013.07.006 PubMed PMID: . 23994223

[57] Teper R, Inzlicht M. Mindful acceptance dampens neuroaffective reactions to external and rewarding performance feedback. Emotion. 2014;14(1):105–14. 10.1037/a0034296 PubMed PMID: . 24098927

[58] Hertz RM, Laurent HK, Laurent SM. Attachment mediates effects of trait mindfulness on stress responses to conflict. Mindfulness. 2014:1–7. doi: 10.1007/s12671-014-0281-7.

[59] Drabant EM, McRae K, Manuck SB, Hariri AR, Gross JJ. Individual differences in typical reappraisal use predict amygdala and prefrontal responses. Biol Psychiatry. 2009;65(5):367–73. 10.1016/j.biopsych.2008.09.007 PubMed PMID: . 18930182 PMC2855682

[60] Modinos G, Ormel J, Aleman A. Individual differences in dispositional mindfulness and brain activity involved in reappraisal of emotion. Soc Cogn Affect Neurosci. 2010;5(4):369–77. 10.1093/scan/nsq006 PubMed PMID: . 20147457 PMC2999757

[61] Creswell JD, Way BM, Eisenberger NI, Lieberman MD. Neural correlates of dispositional mindfulness during affect labeling. Psychosom Med. 2007;69(6):560–5. 10.1097/PSY.0b013e3180f6171f PubMed PMID: . 17634566

