# Supplementary material for: Self-Reported Trait Mindfulness and Affective Reactivity: A Motivational Approach Using Multiple Psychophysiological Measures
Source: PLoS One. 2015 Mar 6;10(3):e0119466. doi: 10.1371/journal.pone.0119466 (PMC4352075; doi:10.1371/journal.pone.0119466)
Supplement: S1 Table — (DOCX) [file pone.0119466.s001.docx]

**Table S1. IAPS picture codes**

| **Pleasant** | **Neutral** | **Unpleasant** |
| --- | --- | --- |
| 1440 | 2002 | 2710 |
| 1610 | 2020 | 3000 |
| 1920 | 2102 | 3030 |
| 2040 | 2107 | 3053 |
| 2045 | 2200 | 3059 |
| 2057 | 2214 | 3071 |
| 2058 | 2215 | 3100 |
| 2070 | 2305 | 3120 |
| 2080 | 2357 | 3131 |
| 2150 | 2372 | 3140 |
| 2260 | 2441 | 3170 |
| 4220 | 2484 | 3190 |
| 4290 | 2488 | 3195 |
| 4490 | 2493 | 3400 |
| 4611 | 2506 | 3550 |
| 4658 | 2512 | 6370 |
| 4660 | 2513 | 9040 |
| 4668 | 5500 | 9042 |
| 4680 | 5530 | 9043 |
| 4687 | 5531 | 9140 |
| 4690 | 5532 | 9302 |
| 4694 | 5534 | 9322 |
| 4695 | 8465 | 9405 |
| 4800 | 9070 | 9412 |
