# Supplementary material for: Self-Reported Trait Mindfulness and Affective Reactivity: A Motivational Approach Using Multiple Psychophysiological Measures
Source: PLoS One. 2015 Mar 6;10(3):e0119466. doi: 10.1371/journal.pone.0119466 (PMC4352075; doi:10.1371/journal.pone.0119466)
Supplement: S1 Dataset — (ZIP) [file pone.0119466.s002.zip › FILE_S1 file info.pdf]

DISPLAY DICTIONARY.

File Information

[DataSet1] C:\Users\SWIENS\Documents\Dropbox\Dani's Thesis\Paper Drafts\data and figures\Data\PLOSONE_final.sav

Variable Information

Variable	Position	Label	Measurement Level	Role
ID	1	Subject ID	Scale	Input
Sex	2	Sex	Nominal	Input
Age	3	Age (years)	Scale	Input
Education	4	Education (years)	Scale	Input
Handedness	5	Handedness	Nominal	Input
Vision	6	Vision	Nominal	Input
Meditation1	7	Meditation practice	Nominal	Input
Meditation2	8	Meditation practice (years)	Nominal	Input
Meditation3	9	Meditation practice (hrs/wk)	Nominal	Input
FFMQ_1	10	Five Facet Mindfulness Questionnaire	Nominal	Input
FFMQ_2	11	Five Facet Mindfulness Questionnaire	Nominal	Input
FFMQ_3	12	Five Facet Mindfulness Questionnaire (R)	Nominal	Input
FFMQ_4	13	Five Facet Mindfulness Questionnaire	Nominal	Input
FFMQ_5	14	Five Facet Mindfulness Questionnaire (R)	Nominal	Input
FFMQ_6	15	Five Facet Mindfulness Questionnaire	Nominal	Input

Variable Information

Variable	Column Width	Alignment	Print Format	Write Format
ID	8	Right	F5	F5
Sex	8	Right	F5	F5
Age	8	Right	F5	F5
Education	8	Right	F5	F5
Handedness	8	Right	F5	F5
Vision	8	Right	F5	F5
Meditation1	8	Right	F5	F5
Meditation2	8	Right	F5	F5
Meditation3	8	Right	F5	F5
FFMQ_1	5	Right	F2	F2
FFMQ_2	5	Right	F1	F1
FFMQ_3	5	Right	F2	F2
FFMQ_4	5	Right	F2	F2
FFMQ_5	5	Right	F1	F1
FFMQ_6	5	Right	F1	F1

Variable Information

Variable	Position	Label	Measurement Level	Role
FFMQ_7	16	Five Facet Mindfulness Questionnaire	Nominal	Input
FFMQ_8	17	Five Facet Mindfulness Questionnaire (R)	Nominal	Input
FFMQ_9	18	Five Facet Mindfulness Questionnaire	Nominal	Input
FFMQ_10	19	Five Facet Mindfulness Questionnaire (R)	Nominal	Input
FFMQ_11	20	Five Facet Mindfulness Questionnaire	Nominal	Input
FFMQ_12	21	Five Facet Mindfulness Questionnaire (R)	Nominal	Input
FFMQ_13	22	Five Facet Mindfulness Questionnaire (R)	Nominal	Input
FFMQ_14	23	Five Facet Mindfulness Questionnaire (R)	Nominal	Input
FFMQ_15	24	Five Facet Mindfulness Questionnaire	Nominal	Input
FFMQ_16	25	Five Facet Mindfulness Questionnaire (R)	Nominal	Input
FFMQ_17	26	Five Facet Mindfulness Questionnaire (R)	Nominal	Input
FFMQ_18	27	Five Facet Mindfulness Questionnaire (R)	Nominal	Input

Variable Information

Variable	Column Width	Alignment	Print Format	Write Format
FFMQ_7	5	Right	F1	F1
FFMQ_8	5	Right	F1	F1
FFMQ_9	5	Right	F2	F2
FFMQ_10	5	Right	F1	F1
FFMQ_11	5	Right	F1	F1
FFMQ_12	5	Right	F1	F1
FFMQ_13	5	Right	F1	F1
FFMQ_14	5	Right	F1	F1
FFMQ_15	5	Right	F1	F1
FFMQ_16	5	Right	F1	F1
FFMQ_17	5	Right	F2	F2
FFMQ_18	5	Right	F1	F1

Variable Information

Variable	Position	Label	Measurement Level	Role
FFMQ_19	28	Five Facet Mindfulness Questionnaire	Nominal	Input
FFMQ_20	29	Five Facet Mindfulness Questionnaire	Nominal	Input
FFMQ_21	30	Five Facet Mindfulness Questionnaire	Nominal	Input
FFMQ_22	31	Five Facet Mindfulness Questionnaire (R)	Nominal	Input
FFMQ_23	32	Five Facet Mindfulness Questionnaire (R)	Nominal	Input
FFMQ_24	33	Five Facet Mindfulness Questionnaire	Nominal	Input
FFMQ_25	34	Five Facet Mindfulness Questionnaire (R)	Nominal	Input
FFMQ_26	35	Five Facet Mindfulness Questionnaire	Nominal	Input
FFMQ_27	36	Five Facet Mindfulness Questionnaire	Nominal	Input
FFMQ_28	37	Five Facet Mindfulness Questionnaire (R)	Nominal	Input
FFMQ_29	38	Five Facet Mindfulness Questionnaire	Nominal	Input
FFMQ_30	39	Five Facet Mindfulness Questionnaire (R)	Nominal	Input

Variable Information

Variable	Column Width	Alignment	Print Format	Write Format
FFMQ_19	5	Right	F2	F2
FFMQ_20	5	Right	F1	F1
FFMQ_21	5	Right	F2	F2
FFMQ_22	5	Right	F1	F1
FFMQ_23	5	Right	F1	F1
FFMQ_24	5	Right	F2	F2
FFMQ_25	5	Right	F1	F1
FFMQ_26	5	Right	F1	F1
FFMQ_27	5	Right	F1	F1
FFMQ_28	5	Right	F1	F1
FFMQ_29	5	Right	F2	F2
FFMQ_30	5	Right	F1	F1

Variable Information

Variable	Position	Label	Measurement Level	Role
FFMQ_31	40	Five Facet Mindfulness Questionnaire	Nominal	Input
FFMQ_32	41	Five Facet Mindfulness Questionnaire	Nominal	Input
FFMQ_33	42	Five Facet Mindfulness Questionnaire	Nominal	Input
FFMQ_34	43	Five Facet Mindfulness Questionnaire (R)	Nominal	Input
FFMQ_35	44	Five Facet Mindfulness Questionnaire (R)	Nominal	Input
FFMQ_36	45	Five Facet Mindfulness Questionnaire	Nominal	Input
FFMQ_37	46	Five Facet Mindfulness Questionnaire	Nominal	Input
FFMQ_38	47	Five Facet Mindfulness Questionnaire (R)	Nominal	Input
FFMQ_39	48	Five Facet Mindfulness Questionnaire (R)	Nominal	Input
STAIT_1	49	State-Trait Anxiety Inventory-Trait	Nominal	Input
STAIT_2	50	State-Trait Anxiety Inventory-Trait	Nominal	Input
STAIT_3	51	State-Trait Anxiety Inventory-Trait	Nominal	Input

Variable Information

Variable	Column Width	Alignment	Print Format	Write Format
FFMQ_31	5	Right	F1	F1
FFMQ_32	5	Right	F1	F1
FFMQ_33	5	Right	F2	F2
FFMQ_34	5	Right	F1	F1
FFMQ_35	5	Right	F1	F1
FFMQ_36	5	Right	F1	F1
FFMQ_37	5	Right	F2	F2
FFMQ_38	5	Right	F1	F1
FFMQ_39	5	Right	F2	F2
STAIT_1	5	Right	F1	F1
STAIT_2	5	Right	F1	F1
STAIT_3	5	Right	F1	F1

Variable Information

Variable	Position	Label	Measurement Level	Role
STAIT_4	52	State-Trait Anxiety Inventory-Trait	Nominal	Input
STAIT_5	53	State-Trait Anxiety Inventory-Trait	Nominal	Input
STAIT_6	54	State-Trait Anxiety Inventory-Trait	Nominal	Input
STAIT_7	55	State-Trait Anxiety Inventory-Trait	Nominal	Input
STAIT_8	56	State-Trait Anxiety Inventory-Trait	Nominal	Input
STAIT_9	57	State-Trait Anxiety Inventory-Trait	Nominal	Input
STAIT_10	58	State-Trait Anxiety Inventory-Trait	Nominal	Input
STAIT_11	59	State-Trait Anxiety Inventory-Trait	Nominal	Input
STAIT_12	60	State-Trait Anxiety Inventory-Trait	Nominal	Input
STAIT_13	61	State-Trait Anxiety Inventory-Trait	Nominal	Input
STAIT_14	62	State-Trait Anxiety Inventory-Trait	Nominal	Input
STAIT_15	63	State-Trait Anxiety Inventory-Trait	Nominal	Input

Variable Information

Variable	Column Width	Alignment	Print Format	Write Format
STAIT_4	5	Right	F1	F1
STAIT_5	5	Right	F1	F1
STAIT_6	5	Right	F1	F1
STAIT_7	5	Right	F1	F1
STAIT_8	5	Right	F1	F1
STAIT_9	5	Right	F1	F1
STAIT_10	5	Right	F1	F1
STAIT_11	5	Right	F1	F1
STAIT_12	5	Right	F1	F1
STAIT_13	5	Right	F1	F1
STAIT_14	5	Right	F1	F1
STAIT_15	5	Right	F1	F1

Variable Information

Variable	Position	Label	Measurement Level	Role
STAIT_16	64	State-Trait Anxiety Inventory-Trait	Nominal	Input
STAIT_17	65	State-Trait Anxiety Inventory-Trait	Nominal	Input
STAIT_18	66	State-Trait Anxiety Inventory-Trait	Nominal	Input
STAIT_19	67	State-Trait Anxiety Inventory-Trait	Nominal	Input
STAIT_20	68	State-Trait Anxiety Inventory-Trait	Nominal	Input
MAAS_1	69	Mindful Attention Awareness Scale	Nominal	Input
MAAS_2	70	Mindful Attention Awareness Scale	Nominal	Input
MAAS_3	71	Mindful Attention Awareness Scale	Nominal	Input
MAAS_4	72	Mindful Attention Awareness Scale	Nominal	Input
MAAS_5	73	Mindful Attention Awareness Scale	Nominal	Input
MAAS_6	74	Mindful Attention Awareness Scale	Nominal	Input
MAAS_7	75	Mindful Attention Awareness Scale	Nominal	Input

Variable Information

Variable	Column Width	Alignment	Print Format	Write Format
STAIT_16	5	Right	F1	F1
STAIT_17	5	Right	F1	F1
STAIT_18	5	Right	F1	F1
STAIT_19	5	Right	F1	F1
STAIT_20	5	Right	F1	F1
MAAS_1	5	Right	F2	F2
MAAS_2	5	Right	F1	F1
MAAS_3	5	Right	F1	F1
MAAS_4	5	Right	F1	F1
MAAS_5	5	Right	F1	F1
MAAS_6	5	Right	F2	F2
MAAS_7	5	Right	F1	F1

Variable Information

Variable	Position	Label	Measurement Level	Role
MAAS_8	76	Mindful Attention Awareness Scale	Nominal	Input
MAAS_9	77	Mindful Attention Awareness Scale	Nominal	Input
MAAS_10	78	Mindful Attention Awareness Scale	Nominal	Input
MAAS_11	79	Mindful Attention Awareness Scale	Nominal	Input
MAAS_12	80	Mindful Attention Awareness Scale	Nominal	Input
MAAS_13	81	Mindful Attention Awareness Scale	Nominal	Input
MAAS_14	82	Mindful Attention Awareness Scale	Nominal	Input
MAAS_15	83	Mindful Attention Awareness Scale	Nominal	Input
BDI1	84	Beck Depression Inventory	Nominal	Input
BDI2	85	Beck Depression Inventory	Nominal	Input
BDI3	86	Beck Depression Inventory	Nominal	Input
BDI4	87	Beck Depression Inventory	Nominal	Input
BDI5	88	Beck Depression Inventory	Nominal	Input
BDI6	89	Beck Depression Inventory	Nominal	Input

Variable Information

Variable	Column Width	Alignment	Print Format	Write Format
MAAS_8	5	Right	F1	F1
MAAS_9	5	Right	F1	F1
MAAS_10	5	Right	F1	F1
MAAS_11	5	Right	F1	F1
MAAS_12	5	Right	F2	F2
MAAS_13	5	Right	F2	F2
MAAS_14	5	Right	F1	F1
MAAS_15	5	Right	F2	F2
BDI1	5	Right	F1	F1
BDI2	5	Right	F1	F1
BDI3	5	Right	F1	F1
BDI4	5	Right	F1	F1
BDI5	5	Right	F1	F1
BDI6	5	Right	F1	F1

Variable Information

Variable	Position	Label	Measurement Level	Role
BDI7	90	Beck Depression Inventory	Nominal	Input
BDI8	91	Beck Depression Inventory	Nominal	Input
BDI9	92	Beck Depression Inventory	Nominal	Input
BDI10	93	Beck Depression Inventory	Nominal	Input
BDI11	94	Beck Depression Inventory	Nominal	Input
BDI12	95	Beck Depression Inventory	Nominal	Input
BDI13	96	Beck Depression Inventory	Nominal	Input
BDI14	97	Beck Depression Inventory	Nominal	Input
BDI15	98	Beck Depression Inventory	Nominal	Input
BDI16	99	Beck Depression Inventory	Nominal	Input
BDI17	100	Beck Depression Inventory	Nominal	Input
BDI18	101	Beck Depression Inventory	Nominal	Input
BDI19	102	Beck Depression Inventory	Nominal	Input
BDI20	103	Beck Depression Inventory	Nominal	Input
BDI21	104	Beck Depression Inventory	Nominal	Input
STAI_T_sum	105	STAI trait sum	Scale	Input
BDI_sum	106	BDI sum score	Nominal	Input
FFMQ_mean	107	FFMQ mean	Scale	Input

Variable Information

Variable	Column Width	Alignment	Print Format	Write Format
BDI7	5	Right	F1	F1
BDI8	5	Right	F1	F1
BDI9	5	Right	F1	F1
BDI10	5	Right	F1	F1
BDI11	5	Right	F1	F1
BDI12	5	Right	F1	F1
BDI13	5	Right	F1	F1
BDI14	5	Right	F1	F1
BDI15	5	Right	F1	F1
BDI16	5	Right	F1	F1
BDI17	5	Right	F1	F1
BDI18	5	Right	F1	F1
BDI19	5	Right	F1	F1
BDI20	5	Right	F1	F1
BDI21	5	Right	F1	F1
STAI_T_sum	12	Right	F8.2	F8.2
BDI_sum	8	Right	F8.2	F8.2
FFMQ_mean	8	Right	F8.2	F8.2

Variable Information

Variable	Position	Label	Measurement Level	Role
FFMQ_AWA_mean	108	FFMQ-Acting with Awareness mean	Scale	Input
FFMQ_D_mean	109	FFMQ-Describing mean	Scale	Input
FFMQ_O_mean	110	FFMQ-Observing mean	Scale	Input
FFMQ_NJ_mean	111	FFMQ-Non-judging mean	Scale	Input
FFMQ_NR_mean	112	FFMQ-Nonreacting mean	Scale	Input
MAAS_mean	113	MAAS mean	Scale	Input
FFMQ_center	114	FFMQ centered mean	Nominal	Input
FFMQ_AWA_center	115	FFMQ-Acting with Awareness centered mean	Nominal	Input
FFMQ_D_center	116	FFMQ-Describing centered mean	Nominal	Input
FFMQ_O_center	117	FFMQ-Observing centered mean	Nominal	Input
FFMQ_NJ_center	118	FFMQ-Non-judging centered mean	Nominal	Input
FFMQ_NR_center	119	FFMQ-Nonreacting centered mean	Nominal	Input
MAAS_center	120	MAAS centered mean	Nominal	Input
FFMQ_NR_di	121	FFMQ-Nonreactivity median split (26 low and 25 high)	Nominal	Input

Variable Information

Variable	Column Width	Alignment	Print Format	Write Format
FFMQ_AWA_mean	8	Right	F8.2	F8.2
FFMQ_D_mean	8	Right	F8.2	F8.2
FFMQ_O_mean	8	Right	F8.2	F8.2
FFMQ_NJ_mean	8	Right	F8.2	F8.2
FFMQ_NR_mean	11	Right	F8.2	F8.2
MAAS_mean	8	Right	F8.2	F8.2
FFMQ_center	8	Right	F8.2	F8.2
FFMQ_AWA_center	8	Right	F8.2	F8.2
FFMQ_D_center	8	Right	F8.2	F8.2
FFMQ_O_center	8	Right	F8.2	F8.2
FFMQ_NJ_center	8	Right	F8.2	F8.2
FFMQ_NR_center	8	Right	F8.2	F8.2
MAAS_center	8	Right	F8.2	F8.2
FFMQ_NR_di	8	Right	F8	F8

Variable Information

Variable	Position	Label	Measurement Level	Role
EMG_N_valid_trials	122	EMG, number of valid (even zero) trials (max = 72)	Scale	Input
EMG_Perc_null_responses	123	EMG, percent null responses of all valid trials	Scale	Input
EMG_n_outliers	124	EMG, number of outliers (x > +/-3 SDs)	Nominal	Input
EMG_min_n_cond	125	EMG, minimum number of trials (max = 8 trials) for any of the conditions	Nominal	Input
EMGPos1_magnitude	126	EMG magnitude to positive pics, startle 1	Scale	Input
EMGPos2_magnitude	127	EMG magnitude to positive pics, startle 2	Scale	Input
EMGPos3_magnitude	128	EMG magnitude to positive pics, startle 3	Scale	Input
EMGNeu1_magnitude	129	EMG magnitude to neutral pics, startle 1	Scale	Input
EMGNeu2_magnitude	130	EMG magnitude to neutral pics, startle 2	Scale	Input
EMGNeu3_magnitude	131	EMG magnitude to neutral pics, startle 3	Scale	Input
EMGNeg1_magnitude	132	EMG magnitude to negative pics, startle 1	Scale	Input
EMGNeg2_magnitude	133	EMG magnitude to negative pics, startle 2	Scale	Input

Variable Information

Variable	Column Width	Alignment	Print Format	Write Format
EMG_N_valid_trials	8	Right	F8	F8
EMG_Perc_null_responses	8	Right	F8.2	F8.2
EMG_n_outliers	8	Right	F8	F8
EMG_min_n_cond	8	Right	F8	F8
EMGPos1_magnitude	8	Right	F8.2	F8.2
EMGPos2_magnitude	8	Right	F8.2	F8.2
EMGPos3_magnitude	8	Right	F8.2	F8.2
EMGNeu1_magnitude	8	Right	F8.2	F8.2
EMGNeu2_magnitude	8	Right	F8.2	F8.2
EMGNeu3_magnitude	8	Right	F8.2	F8.2
EMGNeg1_magnitude	8	Right	F8.2	F8.2
EMGNeg2_magnitude	8	Right	F8.2	F8.2

Variable Information

Variable	Position	Label	Measurement Level	Role
EMGNeg3_magnitude	134	EMG magnitude to negative pics, startle 3	Scale	Input
SCRPicpos_ntrials	135	SCR to positive pictures, number of trials	Scale	Input
SCRPicneu_ntrials	136	SCR to neutral pictures, number of trials	Scale	Input
SCRPicneg_ntrials	137	SCR to negative pictures, number of trials	Scale	Input
SCRStlpos_ntrials	138	SCR startles on positive pictures, number of trials	Nominal	Input
SCRStlneu_ntrials	139	SCR startles on neutral pictures, number of trials	Nominal	Input
SCRStlneg_ntrials	140	SCR startles on negative pictures, number of trials	Nominal	Input
SCRPicpos_magnitude	141	SCR magnitude to positive pictures	Scale	Input
SCRPicneu_magnitude	142	SCR magnitude to neutral pictures	Scale	Input
SCRPicneg_magnitude	143	SCR magnitude to negative pictures	Scale	Input

Variable Information

Variable	Column Width	Alignment	Print Format	Write Format
EMGNeg3_magnitude	8	Right	F8.2	F8.2
SCRPicpos_ntrials	8	Right	F8.2	F8.2
SCRPicneu_ntrials	8	Right	F8.2	F8.2
SCRPicneg_ntrials	8	Right	F8.2	F8.2
SCRStlpos_ntrials	8	Right	F8.2	F8.2
SCRStlneu_ntrials	8	Right	F8.2	F8.2
SCRStlneg_ntrials	8	Right	F8.2	F8.2
SCRPicpos_magnitude	8	Right	F8.2	F8.2
SCRPicneu_magnitude	8	Right	F8.2	F8.2
SCRPicneg_magnitude	8	Right	F8.2	F8.2

Variable Information

Variable	Position	Label	Measurement Level	Role
SCRStlpos_magnitude	144	SCR magnitude to startles on positive pictures	Scale	Input
SCRStlneu_magnitude	145	SCR magnitude to startles on neutral pictures	Scale	Input
SCRStlneg_magnitude	146	SCR magnitude to startles on negative pictures	Scale	Input
SAMPlePos	147	Self-Assesment Manikin, valence for positive pictures	Scale	Input
SAMAroPos	148	Self-Assesment Manikin, arousal for positive pictures	Scale	Input
SAMPleNeu	149	Self-Assesment Manikin, valence for neutral pictures	Scale	Input
SAMAroNeu	150	Self-Assesment Manikin, arousal for neutral pictures	Scale	Input
SAMPleNeg	151	Self-Assesment Manikin, valence for negative pictures	Scale	Input
SAMAroNeg	152	Self-Assesment Manikin, arousal for negative pictures	Scale	Input

Variable Information

Variable	Column Width	Alignment	Print Format	Write Format
SCRStlpos_magnitude	8	Right	F8.2	F8.2
SCRStlneu_magnitude	8	Right	F8.2	F8.2
SCRStlneg_magnitude	8	Right	F8.2	F8.2
SAMPlePos	8	Right	F8.2	F8.2
SAMAroPos	8	Right	F8.2	F8.2
SAMPleNeu	8	Right	F8.2	F8.2
SAMAroNeu	8	Right	F8.2	F8.2
SAMPleNeg	8	Right	F8.2	F8.2
SAMAroNeg	8	Right	F8.2	F8.2

Variable Information

Variable	Position	Label	Measurement Level	Role
P3_StlPos1	153	P3 magnitude to positive pics, startle 1	Scale	Input
P3_StlPos2	154	P3 magnitude to positive pics, startle 2	Scale	Input
P3_StlPos3	155	P3 magnitude to positive pics, startle 3	Scale	Input
P3_StlNeu1	156	P3 magnitude to neutral pics, startle 1	Scale	Input
P3_StlNeu2	157	P3 magnitude to neutral pics, startle 2	Scale	Input
P3_StlNeu3	158	P3 magnitude to neutral pics, startle 3	Scale	Input
P3_StlNeg1	159	P3 magnitude to negative pics, startle 1	Scale	Input
P3_StlNeg2	160	P3 magnitude to negative pics, startle 2	Scale	Input
P3_StlNeg3	161	P3 magnitude to negative pics, startle 3	Scale	Input
EPN_PicPos	162	EPN magnitude to positive pictures	Scale	Input
EPN_PicNeu	163	EPN magnitude to neutral pictures	Scale	Input
EPN_PicNeg	164	EPN magnitude to negative pictures	Scale	Input
LPP_PicPos	165	LPP magnitude to positive pictures	Scale	Input
LPP_PicNeu	166	LPP magnitude to neutral pictures	Scale	Input
LPP_PicNeg	167	LPP magnitude to negative pictures	Scale	Input

Variable Information

Variable	Column Width	Alignment	Print Format	Write Format
P3_StlPos1	8	Right	F8.2	F8.2
P3_StlPos2	8	Right	F8.2	F8.2
P3_StlPos3	8	Right	F8.2	F8.2
P3_StlNeu1	8	Right	F8.2	F8.2
P3_StlNeu2	8	Right	F8.2	F8.2
P3_StlNeu3	8	Right	F8.2	F8.2
P3_StlNeg1	8	Right	F8.2	F8.2
P3_StlNeg2	8	Right	F8.2	F8.2
P3_StlNeg3	8	Right	F8.2	F8.2
EPN_PicPos	8	Right	F8.2	F8.2
EPN_PicNeu	8	Right	F8.2	F8.2
EPN_PicNeg	8	Right	F8.2	F8.2
LPP_PicPos	8	Right	F8.2	F8.2
LPP_PicNeu	8	Right	F8.2	F8.2
LPP_PicNeg	8	Right	F8.2	F8.2

Variables in the working file

Variable Values

Value		Label
Sex	1	Male
	2	Female
Handedness	1	Right
	2	Left
Vision	1	Normal
	2	Corrected with glasses or contacts
	3	Other
Meditation1	1	Yes
	2	No
FFMQ_1	1	Never or very rarely true
	2	Rarely true
	3	Sometimes true
	4	Often true
	5	Very often or always true
FFMQ_2	1	Never or very rarely true
	2	Rarely true
	3	Sometimes true
	4	Often true
	5	Very often or always true
FFMQ_3	1	Never or very rarely true
	2	Rarely true
	3	Sometimes true
	4	Often true
	5	Very often or always true
FFMQ_4	1	Never or very rarely true
	2	Rarely true
	3	Sometimes true
	4	Often true
	5	Very often or always true
FFMQ_5	1	Never or very rarely true
	2	Rarely true
	3	Sometimes true
	4	Often true
	5	Very often or always true
FFMQ_6	1	Never or very rarely true
	2	Rarely true
	3	Sometimes true
	4	Often true
	5	Very often or always true
FFMQ_7	1	Never or very rarely true
	2	Rarely true
	3	Sometimes true

Variable Values

Value		Label
	4	Often true
	5	Very often or always true
FFMQ_8	1	Never or very rarely true
	2	Rarely true
	3	Sometimes true
	4	Often true
	5	Very often or always true
FFMQ_9	1	Never or very rarely true
	2	Rarely true
	3	Sometimes true
	4	Often true
	5	Very often or always true
FFMQ_10	1	Never or very rarely true
	2	Rarely true
	3	Sometimes true
	4	Often true
	5	Very often or always true
FFMQ_11	1	Never or very rarely true
	2	Rarely true
	3	Sometimes true
	4	Often true
	5	Very often or always true
FFMQ_12	1	Never or very rarely true
	2	Rarely true
	3	Sometimes true
	4	Often true
	5	Very often or always true
FFMQ_13	1	Never or very rarely true
	2	Rarely true
	3	Sometimes true
	4	Often true
	5	Very often or always true
FFMQ_14	1	Never or very rarely true
	2	Rarely true
	3	Sometimes true
	4	Often true
	5	Very often or always true
FFMQ_15	1	Never or very rarely true
	2	Rarely true
	3	Sometimes true
	4	Often true
	5	Very often or always true

Variable Values

Value		Label
FFMQ_16	1	Never or very rarely true
	2	Rarely true
	3	Sometimes true
	4	Often true
	5	Very often or always true
FFMQ_17	1	Never or very rarely true
	2	Rarely true
	3	Sometimes true
	4	Often true
	5	Very often or always true
FFMQ_18	1	Never or very rarely true
	2	Rarely true
	3	Sometimes true
	4	Often true
	5	Very often or always true
FFMQ_19	1	Never or very rarely true
	2	Rarely true
	3	Sometimes true
	4	Often true
	5	Very often or always true
FFMQ_20	1	Never or very rarely true
	2	Rarely true
	3	Sometimes true
	4	Often true
	5	Very often or always true
FFMQ_21	1	Never or very rarely true
	2	Rarely true
	3	Sometimes true
	4	Often true
	5	Very often or always true
FFMQ_22	1	Never or very rarely true
	2	Rarely true
	3	Sometimes true
	4	Often true
	5	Very often or always true
FFMQ_23	1	Never or very rarely true
	2	Rarely true
	3	Sometimes true
	4	Often true
	5	Very often or always true
FFMQ_24	1	Never or very rarely true
	2	Rarely true
	3	Sometimes true

Variable Values

Value		Label
	4	Often true
	5	Very often or always true
FFMQ_25	1	Never or very rarely true
	2	Rarely true
	3	Sometimes true
	4	Often true
	5	Very often or always true
FFMQ_26	1	Never or very rarely true
	2	Rarely true
	3	Sometimes true
	4	Often true
	5	Very often or always true
FFMQ_27	1	Never or very rarely true
	2	Rarely true
	3	Sometimes true
	4	Often true
	5	Very often or always true
FFMQ_28	1	Never or very rarely true
	2	Rarely true
	3	Sometimes true
	4	Often true
	5	Very often or always true
FFMQ_29	1	Never or very rarely true
	2	Rarely true
	3	Sometimes true
	4	Often true
	5	Very often or always true
FFMQ_30	1	Never or very rarely true
	2	Rarely true
	3	Sometimes true
	4	Often true
	5	Very often or always true
FFMQ_31	1	Never or very rarely true
	2	Rarely true
	3	Sometimes true
	4	Often true
	5	Very often or always true
FFMQ_32	1	Never or very rarely true
	2	Rarely true
	3	Sometimes true
	4	Often true
	5	Very often or always true

Variable Values

Value		Label
FFMQ_33	1	Never or very rarely true
	2	Rarely true
	3	Sometimes true
	4	Often true
	5	Very often or always true
FFMQ_34	1	Never or very rarely true
	2	Rarely true
	3	Sometimes true
	4	Often true
	5	Very often or always true
FFMQ_35	1	Never or very rarely true
	2	Rarely true
	3	Sometimes true
	4	Often true
	5	Very often or always true
FFMQ_36	1	Never or very rarely true
	2	Rarely true
	3	Sometimes true
	4	Often true
	5	Very often or always true
FFMQ_37	1	Never or very rarely true
	2	Rarely true
	3	Sometimes true
	4	Often true
	5	Very often or always true
FFMQ_38	1	Never or very rarely true
	2	Rarely true
	3	Sometimes true
	4	Often true
	5	Very often or always true
FFMQ_39	1	Never or very rarely true
	2	Rarely true
	3	Sometimes true
	4	Often true
	5	Very often or always true
STAIT_1	1	Almost never
	2	Sometimes
	3	Often
	4	Almost always
STAIT_2	1	Almost never
	2	Sometimes
	3	Often
	4	Almost always

Variable Values

Value		Label
STAIT_3	1	Almost never
	2	Sometimes
	3	Often
	4	Almost always
STAIT_4	1	Almost never
	2	Sometimes
	3	Often
	4	Almost always
STAIT_5	1	Almost never
	2	Sometimes
	3	Often
	4	Almost always
STAIT_6	1	Almost never
	2	Sometimes
	3	Often
	4	Almost always
STAIT_7	1	Almost never
	2	Sometimes
	3	Often
	4	Almost always
STAIT_8	1	Almost never
	2	Sometimes
	3	Often
	4	Almost always
STAIT_9	1	Almost never
	2	Sometimes
	3	Often
	4	Almost always
STAIT_10	1	Almost never
	2	Sometimes
	3	Often
	4	Almost always
STAIT_11	1	Almost never
	2	Sometimes
	3	Often
	4	Almost always
STAIT_12	1	Almost never
	2	Sometimes
	3	Often
	4	Almost always
STAIT_13	1	Almost never
	2	Sometimes

Variable Values

Value		Label
	3	Often
	4	Almost always
STAIT_14	1	Almost never
	2	Sometimes
	3	Often
	4	Almost always
STAIT_15	1	Almost never
	2	Sometimes
	3	Often
	4	Almost always
STAIT_16	1	Almost never
	2	Sometimes
	3	Often
	4	Almost always
STAIT_17	1	Almost never
	2	Sometimes
	3	Often
	4	Almost always
STAIT_18	1	Almost never
	2	Sometimes
	3	Often
	4	Almost always
STAIT_19	1	Almost never
	2	Sometimes
	3	Often
	4	Almost always
STAIT_20	1	Almost never
	2	Sometimes
	3	Often
	4	Almost always
MAAS_1	1	Almost always
	2	Very frequently
	3	Somewhat frequently
	4	Somewhat infrequently
	5	Very infrequently
	6	Almost never
MAAS_2	1	Almost always
	2	Very frequently
	3	Somewhat frequently
	4	Somewhat infrequently
	5	Very infrequently
	6	Almost never

Variable Values

Value		Label
MAAS_3	1	Almost always
	2	Very frequently
	3	Somewhat frequently
	4	Somewhat infrequently
	5	Very infrequently
	6	Almost never
MAAS_4	1	Almost always
	2	Very frequently
	3	Somewhat frequently
	4	Somewhat infrequently
	5	Very infrequently
	6	Almost never
MAAS_5	1	Almost always
	2	Very frequently
	3	Somewhat frequently
	4	Somewhat infrequently
	5	Very infrequently
	6	Almost never
MAAS_6	1	Almost always
	2	Very frequently
	3	Somewhat frequently
	4	Somewhat infrequently
	5	Very infrequently
	6	Almost never
MAAS_7	1	Almost always
	2	Very frequently
	3	Somewhat frequently
	4	Somewhat infrequently
	5	Very infrequently
	6	Almost never
MAAS_8	1	Almost always
	2	Very frequently
	3	Somewhat frequently
	4	Somewhat infrequently
	5	Very infrequently
	6	Almost never
MAAS_9	1	Almost always
	2	Very frequently
	3	Somewhat frequently
	4	Somewhat infrequently
	5	Very infrequently
	6	Almost never

Variable Values

Value		Label
MAAS_10	1	Almost always
	2	Very frequently
	3	Somewhat frequently
	4	Somewhat infrequently
	5	Very infrequently
	6	Almost never
MAAS_11	1	Almost always
	2	Very frequently
	3	Somewhat frequently
	4	Somewhat infrequently
	5	Very infrequently
	6	Almost never
MAAS_12	1	Almost always
	2	Very frequently
	3	Somewhat frequently
	4	Somewhat infrequently
	5	Very infrequently
	6	Almost never
MAAS_13	1	Almost always
	2	Very frequently
	3	Somewhat frequently
	4	Somewhat infrequently
	5	Very infrequently
	6	Almost never
MAAS_14	1	Almost always
	2	Very frequently
	3	Somewhat frequently
	4	Somewhat infrequently
	5	Very infrequently
	6	Almost never
MAAS_15	1	Almost always
	2	Very frequently
	3	Somewhat frequently
	4	Somewhat infrequently
	5	Very infrequently
	6	Almost never
BDI1	1	I do not feel sad.
	2	I feel sad.
	3	I am sad all the time and I can't snap out of it.
	4	I am so sad and unhappy that I can't stand it.

Variable Values

Value		Label
BDI2	1	I am not particularly discouraged about the future.
	2	I feel discouraged about the future.
	3	I feel I have nothing to look forward to.
	4	I feel the future is hopeless and that things cannot improve.
BDI3	1	I do not feel like a failure.
	2	I feel I have failed more than the average person.
	3	As I look back on my life, all I can see is a lot of failures.
	4	I feel I am a complete failure as a person.
BDI4	1	I get as much satisfaction out of things as I used to.
	2	I don't enjoy things the way I used to.
	3	I don't get real satisfaction out of anything anymore.
	4	I am dissatisfied or bored with everything.
BDI5	1	I don't feel particularly guilty.
	2	I feel guilty a good part of the time.
	3	I feel quite guilty most of the time.
	4	I feel guilty all of the time.
BDI6	1	I don't feel I am being punished.
	2	I feel I may be punished.
	3	I expect to be punished.
	4	I feel I am being punished.
BDI7	1	I don't feel disappointed in myself.
	2	I am disappointed in myself.
	3	I am disgusted with myself.
	4	I hate myself.
BDI8	1	I don't feel I am any worse than anybody else.
	2	I am critical of myself for my weaknesses or mistakes.
	3	I blame myself all the time for my faults.
	4	I blame myself for everything bad that happens.
BDI9	1	I don't have any thoughts of killing myself.
	2	I have thoughts of killing myself, but I would not carry them out.

Variable Values

Value		Label
	3	I would like to kill myself.
	4	I would kill myself if I had the chance.
BDI10	1	I don't cry any more than usual.
	2	I cry more now than I used to.
	3	I cry all the time now.
	4	I used to be able to cry, but now I can't cry even though I want to.
BDI11	1	I am no more irritated by things than I ever was.
	2	I am slightly more irritated now than usual.
	3	I am quite annoyed or irritated a good deal of the time.
	4	I feel irritated all the time.
BDI12	1	I have not lost interest in other people.
	2	I am less interested in other people than I used to be.
	3	I have lost most of my interest in other people.
	4	I have lost all of my interest in other people.
BDI13	1	I make decisions about as well as I ever could.
	2	I put off making decisions more than I used to.
	3	I have greater difficulty in making decisions more than I used to.
	4	I can't make decisions at all anymore.
BDI14	1	I don't feel that I look any worse than I used to.
	2	I am worried that I am looking old or unattractive.
	3	I feel there are permanent changes in my appearance that make me look unattractive.
	4	I believe that I look ugly.
BDI15	1	I can work about as well as before.
	2	It takes an extra effort to get started at doing something.
	3	I have to push myself very hard to do anything.
	4	I can't do any work at all.
BDI16	1	I can sleep as well as usual.
	2	I don't sleep as well as I used to.

Variable Values

Value		Label
	3	I wake up 12 hours earlier than usual and find it hard to get back to sleep.
	4	I wake up several hours earlier than I used to and cannot get back to sleep.
BDI17	1	I don't get more tired than usual.
	2	I get tired more easily than I used to.
	3	I get tired from doing almost anything.
	4	I am too tired to do anything.
BDI18	1	My appetite is no worse than usual.
	2	My appetite is not as good as it used to be.
	3	My appetite is much worse now.
	4	I have no appetite at all anymore.
BDI19	1	I haven't lost much weight, if any, lately.
	2	I have lost more than five pounds.
	3	I have lost more than ten pounds.
	4	I have lost more than fifteen pounds.
BDI20	1	I am no more worried about my health than usual.
	2	I am worried about physical problems like aches, pains, upset stomach, or constipation.
	3	I am very worried about physical problems and it's hard to think of much else.
	4	I am so worried about my physical problems that I cannot think of anything else.
BDI21	1	I have not noticed any recent change in my interest in sex.
	2	I am less interested in sex than I used to be.
	3	I have almost no interest in sex.
	4	I have lost interest in sex completely.
FFMQ_NR_di	1	high
	2	low
